# Unveiling mysteries of aging: the potential of melatonin in preventing neurodegenerative diseases in older adults

**DOI:** 10.1007/s10522-025-10254-7

**Published:** 2025-06-24

**Authors:** Omer Unal, Nilufer Akgun-Unal, Abdulkerim Kasim Baltaci

**Affiliations:** 1 Department of Physiology, Medical Faculty, Kirikkale University, Kirikkale, Turkey; 2 Department of Biophysics, Medicine Faculty, Ondokuz Mayis University, Samsun, Turkey; 3 Department of Physiology, Medical Faculty, Selcuk University, Konya, Turkey

**Keywords:** Melatonin, Aging, Neurodegenarative disease, Cholinergic system, Melatonergic system, Melatonin receptors

## Abstract

Neurodegenerative conditions, including Alzheimer’s disease, Parkinson’s disease, and Huntington’s disease, result in a substantial health problem for the elderly, marked by ongoing neuronal degeneration and a deterioration in mental faculties. These disorders are frequently linked to oxidative stress, problems with mitochondria, and persistent inflammation in the brain, which worsen neuronal damage. The neurohormone melatonin, primarily secreted by the pineal gland, has gained recognition as a promising therapeutic agent due to its antioxidant, anti-inflammatory, and neuroprotective effects. Melatonin’s functions extend beyond its regulation of circadian rhythms, as research has demonstrated its ability to remove free radicals, improve mitochondrial performance, and adjust immune system responses, ultimately reducing the progression of neurodegenerative disease. Research findings from preclinical and clinical trials imply that taking melatonin supplements could lead to improved cognitive abilities, slower disease progression, and an overall better quality of life for elderly individuals suffering from neurodegenerative conditions. The mechanisms through which melatonin acts, the best dosage, and its long-term effectiveness are still being researched. This review underscores the potential benefits of melatonin as a supplementary treatment for neurodegenerative disorders in older adults, stressing the necessity for additional studies to confirm its efficacy and standardize its use in treatment plans.

## Introduction

The hormone melatonin, or N-acetyl-5-methoxytryptamine, is released during the nighttime hours, primarily serving to inform the body’s physiological processes about the daily light–dark cycle. This component is involved in the regulation of functions that adjust to variations in photoperiod, including circadian and seasonal cycles (Samanta 2022). Melatonin is involved in regulating various bodily functions, such as seasonal reproduction (Talpur et al. 2018), sleep patterns (Xie et al. 2017), immune responses (Bondy and Campbell 2020), the suppression of cancer growth (Su et al. 2017), blood pressure management (Hadi et al. 2019), retinal health (Atacak et al. 2023), the control of natural bodily cycles (Zisapel 2018), emotional state and behavior (Song and Yoon 2025), and neutralizing free radicals (Reina and Martínez 2018). Melatonin exerts its influence in most relevant processes via G-protein coupled membrane receptors like MT1 and MT2 (Nikolaev et al. 2021). Beyond these receptors, there is another binding site that was initially considered to be another membrane-bound receptor (MT3) but was ultimately found to be the enzyme quinone reductase 2 (QR2) (Nosjean et al. 2000). MT1 and MT2 receptors have been identified in different CNS regions, such as the SCN (suprachiasmatic nucleus), cerebral cortex, cerebellar cortex, and midbrain (Ng et al. 2017). Studies in the substantia nigra and amygdala have shown that the density of MT1 and MT2 melatonin receptors is reduced in people with Parkinson’s disease (Adi et al. 2010), suggesting that a disruption of the melatonergic system is a potential factor contributing to the sleep–wake cycle disturbances characteristic of Parkinson’s disease. The rhythmic profile of circulating melatonin is lowered in patients with Parkinson’s disease, especially in those who exhibit excessive daytime sleepiness (Videnovic et al. 2014).

Characteristics of neurodegenerative diseases have been defined as involving common pathophysiological mechanisms resulting in neuronal death, typically consisting of three interconnected processes: glutamate excitotoxicity, free radical-induced nerve damage, and mitochondrial dysfunction (Jurcau 2021). Melatonin is a compound that neutralizes oxygen radicals and protects lipids, leading to its consideration as a possible neuroprotective treatment. Moreover, it exhibits anti-excitatory properties and, at moderate concentrations, presents sedative effects (Ahmed et al. 2021), which offers a protective outcome. The probable loss of melatonin is linked to increased neuronal susceptibility that occurs with aging and disease (Chen et al. 2020a). The pressing need for innovative and effective treatments for these neurodegenerative disorders has been highlighted by the vast number of potential new medications discovered to date, despite the fact that they have not had significant disease-altering impacts. The primary goal of current treatment methods remains centered on symptom alleviation, achieved by making up for the loss of neurotransmitters, such as acetylcholine in Alzheimer’s disease using established cholinesterase inhibitors (ChEIs), and dopamine in Parkinson’s disease through the use of dopamine precursors or agonists; notably, ChEI medications like rivastigmine are also used to address cognitive decline in cases of Parkinson’s disease-related dementia (Reingold et al. 2007). The multiple causes underlying these neurological disorders are thought to be the primary explanation for the absence of a cure up to this point. So far, the FDA has primarily approved treatments for AD that focus on compensating for cholinergic neuron loss using drugs such as inhibitors for acetylcholinesterase (AChE) and butyrylcholinesterase (BChE), including medications like tacrine, donepezil, rivastigmine, and galantamine, as well as the glutamatergic drug memantine, which functions as an N-methyl-D-aspartate (NMDA) receptor antagonist (Albertini et al. 2021). Recently, monoclonal antibodies, such as aducanumab, lecanemab, and donanemab, designed to slow the formation of Aβ aggregates, have received FDA approval (Guiselin et al. 2023), but have yet to be cleared by the EMA due to concerns over their side effects and effectiveness (Hiremathad et al. 2018). Regrettably, prescribed medications can offer temporary symptom relief for mild Alzheimer’s disease cases but fail to halt or slow its progression (Piemontese et al. 2018). Consequently, there are no disease-altering treatments currently available for Alzheimer’s disease management. A multi-target approach is being explored in place of traditional single-target drugs. This strategy involves developing directed ligands that can tackle several key targets associated with Alzheimer’s disease simultaneously. Researchers are working to create single drugs that incorporate multiple pharmacophores, thereby addressing various aspects of AD, such as cholinergic and amyloidogenic processes, oxidative stress, neuroinflammation, metal chelation, and the inhibition of other enzymes that have significant roles in these neurodegenerative processes (Sampietro et al. 2022; Vicente-Zurdo et al. 2022). Indole is a prominent structural motif that can possess a wide range of biological activities and useful drug-like properties. Several synthetic and semi-synthetic therapeutic agents contain this compound (Zhang et al. 2021a, b), as do naturally occurring compounds like bioactive endogenous substances (melatonin, serotonin, tryptophan) and alkaloids with neuroprotective effects found in plants and in neurodegenerative diseases such as AD (Liu et al. 2022). Recent research has taken advantage of the neuroprotective properties of indole-containing compounds such as melatonin, which is reduced in AD patients (Rossotti and Rossotti 1965), by combining the indole structure with other bioactive components (e.g., 8-hydroxyquinolines and tacrine) to create compounds with multiple therapeutic benefits aimed at treating AD (Gans et al. 1996).

In this context, the importance of melatonin, a neurohormone known for its antioxidant activity and protective roles, which has neuroprotective effects in neurodegenerative diseases like AD, encourages us to explore the effects of melatonin, which contains hydroxyl substituent groups, in combination with rivastigmine to enhance antioxidant and metal chelation properties. Here, we also present the initial evaluation of the impact of melatonin on neurodegenerative diseases in the elderly, as well as the connection between the cholinergic and melatonergic systems. In this framework, we discuss the underlying mechanisms of melatonin receptors in relation to neurodegenerative diseases, ageing, and the inhibitors of acetylcholinesterase and butyrylcholinesterase. Additionally, we emphasize the need for a holistic approach in the study of ageing and related diseases/conditions.

### Melatonin

**Fig. 1:**
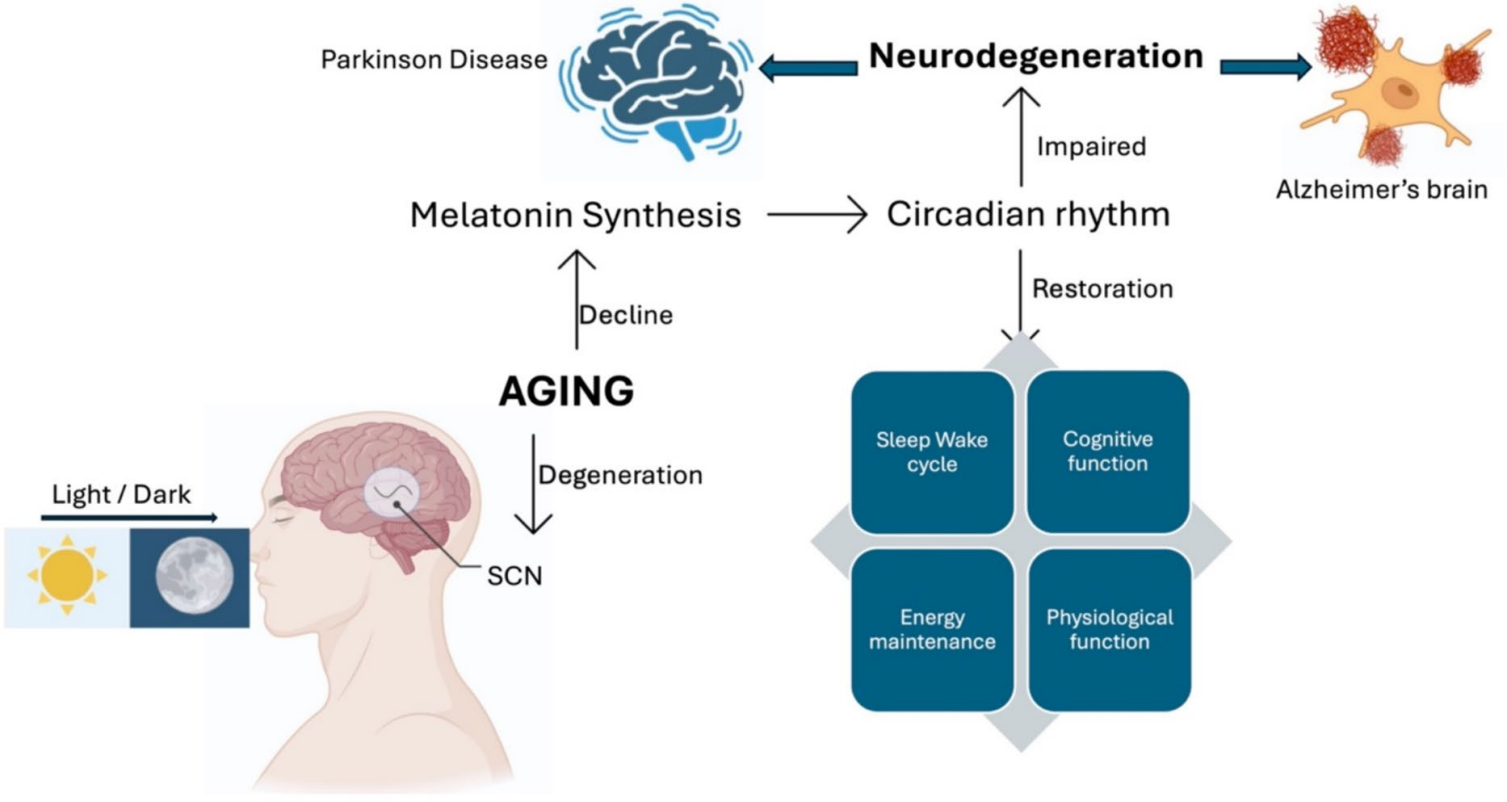
Links between disruption of the body’s natural circadian rhythms and the development of neurodegenerative diseases. Aging and neurodegeneration can disrupt the body’s natural circadian clock, leading to increased neuronal activity, higher production of Aβ, and the accumulation of Lewy bodies. The clearance of pathogenic abnormal proteins may decrease as a result. A number of experts in this field support a two-way connection where the disease’s ongoing progression can influence body clocks, and the disrupted circadian cycles may speed up the disease’s progression. Created with BioRender.com

The pineal gland located in the third ventricle of the brain is responsible for the synthesis of the hormone Melatonin (N-acetyl-methoxy-tryptamine) (MT) (Feng et al. 2023). Lapin and Ebels’ 1976 paper was one of the first to outline these properties, and subsequent studies have provided further clarification on the molecular basis for melatonin’s efficient tissue penetration and cellular accessibility (Lapin and Ebels 1976). Melatonin is also produced outside the brain in lymphocytes, bone marrow, the eyes, and the gastrointestinal system. The synthesized endocrine hormone MLT, a methoxyindole derivative, is known to control human chronobiological functions including circadian rhythms (Bhattacharya et al. 2019). The suprachiasmatic nuclei (SCN), situated in the hypothalamus, are responsible for regulating the physiological circadian rhythm. The SCN triggers melatonin to initiate physiological nighttime functions, including sleep, reduced blood pressure, and metabolism (Zisapel 2018). In essence, the circadian rhythm is an internal biological timekeeper that governs various oscillating processes within a 24-h cycle in the human body (Paulose et al. 2016). 6-Sulfatoxymelatonin, the primary metabolite of endogenous melatonin, plays a crucial role in regulating this rhythm. Besides controlling the central circadian clock, melatonin also regulates the peripheral secretion in various organs and tissues (Albrecht 2012), making melatonin the most reliable indicator of circadian rhythms (Fig. 1). Typically, the nyctohemeral rhythm of this hormone can be forecasted by measuring salivary, urinary sulfatoxymelatonin, and plasma levels. The longer the night, production of melatonin levels increases. Research has demonstrated that increased melatonin levels at night trigger a signal to the body’s organs and cells to regulate their homeostatic metabolic rhythms (Bonmati-Carrion et al. 2014). Melatonin production and circadian rhythms can be substantially impacted by nighttime light, as noted in research by Bonmati-Carrion et al. (2014).

According to research, melatonin can significantly impact the pathways through which estrogen acts on cells, resulting in decreased estrogenic stimulation and potentially producing a beneficial oncostatic effect (Martínez-Campa et al. 2006; Bhattacharya et al. 2019).

Melatonin possesses anti-apoptotic and antioxidant capabilities by neutralizing harmful oxygen derivatives, specifically reactive oxygen species (ROS) (Cong et al. 2023). In addition to ROS, melatonin also neutralizes reactive nitrogen species (RNS), thereby interrupting oxidative and nitrosative damage to macromolecules in all cell compartments. Melatonin is crucial in lowering ROS and RNS levels in mammalian sperm and embryos, which contributes to decreased peroxide concentrations and DNA damage, ultimately leading to an increase in the viability of germ and embryonic cells (Nadri et al. 2024).

Research has discovered that elderly individuals undergo a significant drop in melatonin production, resulting in a tenfold decrease among those in their eighties in comparison to teenagers, which might be associated with age-linked conditions and alterations in metabolism (Martín Giménez et al. 2022; Hardeland 2012). Studies have found that older adults experience a substantial decline in melatonin production, with a tenfold decrease seen in people in their eighties compared to teenagers, which may be linked to age-related conditions and changes in metabolism (Martín Giménez et al. 2022; Hardeland 2012). Studies have found a correlation between low melatonin levels and several health issues, such as dementia, mood disorders, and age-related illnesses, suggesting that reduced melatonin levels may play a role in the onset of these conditions (Hardeland 2012). Evidence from research shows that as individuals grow older, the disruption of melatonin’s natural cycles can have far-reaching consequences, causing further imbalances in the body’s physiological processes (Reiter et al. 2017; Mattis and Sehgal 2016). Additionally, studies have examined how a lack of melatonin affects specific brain functions, such as the regulation of sphingolipid turnover in the hippocampus, which suggests that melatonin is involved in maintaining a balance of lipids, and that this equilibrium may be compromised in aging organisms (Tchekalarova et al. 2025). Overall, the available research indicates that a melatonin deficiency in aging organisms is linked to a range of adverse effects on health and physiological function. There is a scarcity of information regarding specific experimental models and detailed results from studies of organisms deficient in melatonin.

Cyclic nucleotides, for instance, cyclic adenosine monophosphate (cAMP) and cyclic guanosine monophosphate (cGMP), are crucial signal molecules that facilitate a variety of physiological processes, including those affected by melatonin (Tripathy and Bhattamisra 2025; Asma and Marc-André 2022). Melatonin’s effects are mediated through specific receptors, namely MT1 and MT2, which are linked to G-proteins (Chen et al. 2020b). Inhibiting these receptors can result in lower adenylate cyclase activity, consequently reducing cAMP levels. Regulation of sleep and circadian rhythms is reliant on this particular signaling pathway (Xia and Storm 2017; Dubowy and Sehgal 2017). Melatonin can also impact the cGMP pathway, a mechanism that plays a role in several cellular processes, such as vasodilation and neurotransmission. Interactions between melatonin and cGMP signalling pathways could have a function in mood regulation and cognitive function (Rashid et al. 2024). As people age, the sensitivity of melatonin receptors could diminish, which may result in modified cyclic nucleotide signalling (Sarlak et al. 2013). This can lead to reduced physiological responses to melatonin, worsening the consequences of its decrease. Aging causes a significant drop in melatonin levels, which has a substantial impact on sleep patterns, circadian rhythms, and overall well-being. The interaction between melatonin and cyclic nucleotide signalling pathways is vital for comprehending these effects. As melatonin levels decrease, the impact on cyclic nucleotide signalling could lead to several age-related health problems, underscoring melatonin’s role in preserving physiological equilibrium throughout one’s life. Further study of this connection may offer new perspectives on the development of treatments for age-related conditions.

Several senolytic and geroprotective targets are involved in regulating the redox triangle and proteostasis in the process of subcellular aging. The activation of Nrf2 acts as a crucial regulator of antioxidant response, thereby increasing the expression of antioxidant genes, which helps preserve redox balance and offers protection against oxidative stress (He et al. 2020; Verma et al. 2023). Research has found that activating Nrf2 can alleviate oxidative stress and enhance mitochondrial performance in aging models, as indicated by the studies of Yu and Xiao (2021) and Hussain and Kayani (2020). Studies have shown that Nrf2 activators are capable of decreasing age-related deterioration in muscle function and extending lifespan in mice, as reported by Miller et al. (2012; Bruns et al. 2015). Enzymes that rely on NAD + to deacetylate proteins are involved in regulating metabolism, stress responses, and maintaining cellular protein homeostasis. Enhancing mitochondrial function and stimulating autophagy is vital for maintaining protein balance according to research by Xu et al. (2023). Studies have found that melatonin activates SIRT1, resulting in increased mitochondrial biogenesis and a decrease in oxidative stress (Naaz et al. 2020; Akgun-Unal et al. 2023). Verma et al. found that melatonin enhanced the expression of the aging SIRT 1 gene, reduced levels of the neuroinflammatory markers IL-6 and TNF-α, and decreased the neurodegenerative marker Ngb in both young and elderly individuals who received melatonin supplementation and were exposed to artificial nighttime lighting (Verma et al. 2021).Research findings suggest that taking melatonin supplements can enhance cognitive abilities and lower indicators of oxidative harm in older animals (Xu et al. 2019b). Cell growth and nutrient sensing are both influenced by the mTOR signaling pathway. Enhancing autophagy through the suppression of mTOR can lead to improved proteostasis and a decrease in the accumulation of damaged proteins (Fernandes and Demetriades 2021). Studies suggest that mTOR inhibitors, including rapamycin, can prolong lifespan and enhance healthspan in different model species (Zhang et al. 2021b; Bjedov and Rallis 2020). These effects are associated with enhanced autophagy and improved proteostasis. p53tumor suppressor protein is involved in cellular stress responses. Activating the pathway can cause cells to become senescent, yet manipulating it may also stimulate apoptosis in these cells as research by Cerella et al. (2016) found. Research has demonstrated that interfering with the p53 pathway can selectively kill off senescent cells, which in turn improves tissue health and decreases age-related disorders. Studies have shown that senolytics which target p53 are associated with enhanced health results in older mice (van Deursen 2019; Camell et al. 2021). These targets collectively play a role in regulating the redox triangle and proteostasis, underscoring their potential therapeutic roles in ageing and the effects of melatonin.

### Melatonin receptors as therapeutic targets in the central nervous system

Alzheimer’s disease (AD), Parkinson’s disease (PD), and other neurodegenerative diseases (NDs) are widespread among the elderly, with advanced age being the primary risk factor. Worldwide, approximately 55 million individuals are impacted by dementia, with AD responsible for 60–70% of occurrences. The mechanisms driving neurodegenerative disorders encompass oxidative stress, mitochondrial impairment, neuroinflammation, and protein misfolding, such as amyloid-β plaques in AD and α-synuclein in Parkinson’s disease (Lamptey et al. 2022). Melatonin, a hormone governing circadian cycles, has demonstrated potential in countering these mechanisms. This compound exhibits significant antioxidant properties, thereby decreasing oxidative stress and inflammation, and has been associated with enhanced mitochondrial function and the removal of amyloid-β. Studies have shown that giving patients with AD melatonin supplements can lead to improvements in both cognitive function and sleep quality. Long-term studies are scarce, and additional research is required to substantiate the therapeutic effect (Zhang et al. 2025).

#### Sleep disorders

In humans and non-human primates, melatonin treatment in acute form speeds up sleep onset, facilitates its maintenance, or both, and triggers brain waves characteristic of sleep, irrespective of the time of day (Cruz-Sanabria et al. 2023). Melatonin and associated compounds adjust the timing of circadian rhythms by shifting them when administered at specific times that affect the body’s internal clock according to phase response curves that remain consistent across mammals (Burgess et al. 2010). Completely blind people, who cannot perceive light, are able to effectively control their internal body clock by using melatonin (Hartley et al. 2018). Melatonin also helps to adjust the body’s natural sleep–wake cycle in individuals with seasonal affective disorders, leading to a reduction in depression levels (Walker et al. 2020). Melatonin’s distinct effects, resulting from its interactions with melatonin receptors in the central nervous system, are pertinent to the therapeutic objectives of melatonin medications. Difficulty initiating and/or maintaining sleep, as well as sleep that is unrefreshing, are key characteristics of insomnia or sleep–wake disorder, which frequently co-exists with daytime discomfort or emotional distress. A widespread disorder known as insomnia impacts roughly 10% of the global population (Riemann et al. 2022). Current insomnia treatments comprise benzodiazepines and their analogous nonbenzodiazepine medications, which exhibit considerable side effects that lead to impaired cognitive and psychomotor abilities, a heightened risk of falls, rebound, and the potential for dependence or abuse (Laudon and Frydman-Marom 2014). The pursuit of molecules with improved safety profiles has resulted in the creation of a slow-release melatonin formulation (such as Circadin) (Paulis et al. 2012) and artificial melatonin derivatives (including ramelteon, tasimelteon, and agomelatine) (Hardeland 2016). Insomnia is commonly linked to other related health conditions, particularly those affecting mood and the body’s natural circadian rhythms (Laudon and Frydman-Marom 2014). Melatonin and synthetic melatonin agonists typically do not exhibit the side effects (e.g., impairment of learning, memory, or motor function) commonly seen with other sleep medications.

According to the US National Institutes of Health, around 80 million Americans experience symptoms from various circadian rhythm disorders, which can lead to feelings of depression and changes in sleep patterns. Melatonin and melatonin receptor agonists have therapeutic uses in a range of circadian-related conditions, such as jet lag, shift work sleep disorders, delayed sleep phase syndrome, seasonal affective disorder and non-24 h sleep–wake disorder, and major depression (de Bodinat et al. 2010; Zhu and Zee 2012). Studies have shown that the mammalian suprachiasmatic nucleus expresses MT1 and MT2 receptors, as demonstrated by in situ hybridization with 2-[125I]-iodomelatonin and mRNA probes (Hunt et al. 2001). Activation of melatonin receptors decreases neuronal activity in the SCN and parts of the limbic system by stimulating the MT1 receptor, contributing to melatonin’s sleep-inducing effects (Singh et al. 2020).

#### Major depressive disorders

Major depressive disorder stands as the most prevalent mental disorder in the U.S. and is the primary cause of disability impacting approximately 100 million adults globally (Zhao et al. 2024). The condition is marked by a variety of symptoms impacting emotional state, anxiety levels, neurochemical equilibrium, sleep cycles, and circadian and/or seasonal temporal patterns, as well as heightened neurodegenerative changes. The main treatments currently used are tricyclic antidepressants and selective serotonin reuptake inhibitors, which have been found to raise the levels of monoamine neurotransmitters outside the cells. These antidepressants do not address sleep disorders or disturbances related to a person’s natural rhythm and seasonal variations that accompany depressive conditions. Furthermore, prolonged use results in undesirable side effects like weight increase and the combination of cognitive, autonomic, and motor symptoms that characterise the serotonin syndrome (Pannu and Goyal 2024). As a result, researchers are in immediate need to develop antidepressants with new mechanisms of action and decreased side effects.

Melatonin receptors MT1 and MT2 are key targets for the creation of novel antidepressants. Initial evidence suggesting a role for the melatonin receptor in behaviors associated with depression stemmed from the antidepressant-like effects of melatonin and its agonists in rodent studies involving learned helplessness. The antidepressant-like effects of melatonin in the forced swim test were hindered by luzindole, indicating that MT1 and MT2 receptors may be involved (Wang et al. 2022). Melatonin treatment over a long period also increases the growth of new neurons in the hippocampus, a process crucial for the effectiveness of antidepressants in animal studies (Liu et al. 2013; Ramírez-Rodríguez et al. 2009). Mice lacking the MT1 receptor exhibit enhanced depressive-like behavior in a forced swim test (Adamah-Biassi et al. 2014; Weil et al. 2006). The increased presence of MT1 receptor activity in people with major depressive disorder indicates that this receptor type could be a potential target for alleviating some symptoms of depression (Wu et al. 2013). In summary, melatonin agonists may be effective in alleviating symptoms, neurochemical changes, or both associated with clinical manifestations of depressive disorders, primarily through activation of the MT1 receptor.

#### Learning and memory

Changes in the synapses between brain cells store learning and memory (Abraham et al. 2019). Strengthening of the synapse between the two cells occurs when both cells are active simultaneously (Lisman et al. 2018). In 1973, long-term potentiation (LTP)—a mechanism that facilitates the storage of learning and memory in the hippocampus—was first identified by Tim Bliss and Terje Lomo. Research revealed that brief periods of high-frequency stimulation to the hippocampus’ excitatory pathways led to an enduring rise in synaptic excitability, which persisted for even several months, as noted by Rison and Stanton (1995). Further research revealed that LTP is also associated with a shift in ionic flow, resulting in an ionic flow that diverges from that seen during standard synaptic transmission (Morris 2003).

There is a connection between long-term potentiation and memory and learning. Changes in synaptic strength, defined as synaptic plasticity, are believed to be brought about by LTP and are thought to be the mechanism through which learning and memory are stored within the brain. Research findings indicate that synaptic weights are altered following the learning process, thereby establishing a link between learning and the phenomenon of LTP. Changing the mechanisms that underlie synaptic plasticity also alters the rate of learning. Even after the completion of learning, changes to synaptic weights continued to impact the experimental animals’ capacity for recalling what they had learnt. Research has shown that interfering with LTP also disrupts learning and memory functions, highlighting the significant role that LTP plays in these processes (Cantarero et al. 2013; Morris 2003).

Investigations into the hippocampus have been conducted to examine the impact of melatonin. Research findings by Roy et al. (2021) indicate that melatonin alters LTP by modifying synaptic transmission between neurons. A study conducted by Louisa M. Wang found that melatonin suppresses long-term potentiation in neurons. Wang demonstrated this in an experiment involving hippocampal slices of mice. Initially, LTP was triggered using high-frequency stimulation, and the outcome was documented for 60 min. Melatonin was then added to the hippocampal slices, and long-term potentiation was prompted once more. The slopes of field excitatory postsynaptic potential this time were significantly lower, indicating that melatonin had decreased the magnitude of LTP as reported in Wang et al. (2005). Yoshiyuki Takahashi conducted research on melatonin, focusing on its involvement in LTP. He investigated the impact of melatonin on long-term potentiation in the CA1 region of the hippocampus using hippocampal slices from rat brains. Melatonin significantly reduced LTP expression when compared to the control group, as noted by Takahashi and Okada in 2011. Two studies demonstrate that melatonin in the hippocampus acts to prevent LTP. In both experiments, following the addition of melatonin, the levels of LTP were significantly lower than those in the control groups. Melatonin suppresses LTP via the MT2 receptors, as reported by Liu et al. (2016). Previous research on melatonin’s effect on long-term potentiation suggests that melatonin may be impairing the brain’s capacity to form and retain memories. This suppression by the body’s own melatonin, produced by the pineal gland, is an integral component of the body’s natural circadian rhythm. Melatonin and LTP both exhibit a circadian pattern. LTP in the hippocampus is more pronounced during the day when melatonin levels are lower, and less pronounced at night when melatonin levels are higher (Takahashi and Okada 2011). The impact of melatonin on long-term potentiation is contingent upon concentration levels. Research has found that higher levels of melatonin are more effective at suppressing LTP than are lower levels (Shi et al. 2018). The significance of this information becomes apparent when examining the impact of melatonin on cognitive functions such as learning and memory. The natural fluctuation of melatonin levels influences the body’s innate circadian rhythms of learning and memory, which are a typical aspect of bodily function. Taking exogenous melatonin supplements results in higher than typical doses of melatonin entering our bodies, thereby causing a greater-than-normal suppression of LTP. Melatonin supplements may have a severe impact on LTP, thereby causing substantial impairments in an individual’s ability to learn and memory.

Alzheimer’s disease is an irreversible, progressive neurodegenerative condition that is marked by a decline in cognitive function, memory impairment, and irregular behavior patterns (Anwal 2021). Impairments in spatial learning and memory are an important clinical feature of this disease (Sun et al. 2020).Studies have shown that melatonin improves short-term memory in a mouse model of Alzheimer’s disease. Many studies have shown that MT prevents the progression of AD and improves the cognitive impairment associated with the disease through various mechanisms (Shen et al. 2016). For these effects, MT doses need to be approximately twice those required to affect sleep and circadian rhythms (Low et al. 2020). Clinical studies using MT in the range of 50–100 mg/day are needed to investigate the therapeutic validity of MT treatment in AD (Labban et al. 2021a, b). Labban et al. used the highest prophylactic MT dose (80 mg/kg) that did not produce any signs of toxicity in mice in their 2021 study (Labban et al. 2021a, b). They found that MT at this high dose was a potent antioxidant and anti-inflammatory that improved memory outcomes and locomotor activity in an animal model of multiple sclerosis (Abo Taleb and Alghamdi 2020; Alghamdi and AboTaleb 2020).

#### Neuroprotection

Research indicates that melatonin may play a part in shielding against neurodegeneration, apoptosis, and damage from ischemia/reperfusion (Zhang et al. 2024). It is widely accepted that melatonin’s neuroprotective properties are largely due to its ability to neutralize free radicals, as stated in Galano et al. (2013); nonetheless, current research indicates that the activation of MT1 and/or MT2 melatonin receptors may also be a contributing factor. Melatonin has been shown to decrease reactive oxygen species to nearly normal levels in hippocampal slice cultures lacking oxygen and glucose, a reduction that is prevented by luzindole as indicated in a study by Parada et al. (2014). The activity of melatonin receptors may be associated with the activation of antioxidant genes, such as superoxide dismutase and catalase, through a process initiated by receptor-mediated transcriptional regulation. Melatonin receptors could serve as potential targets for new treatments designed to combat the oxidative stress aspects of neuroinflammatory processes.

Melatonin blocks the pathways that lead to mitochondrial cell death in a laboratory model of Huntington’s disease that has a genetic mutation affecting striatal cells. Melatonin also prevents cell death in both cell lines and cultures of primary cerebrocortical and primary striatal neurons, a protective effect that is counteracted by luzindole (Wang et al. 2011). The effect is probably caused by the MT1 receptor, since blocking it makes neurons in culture more prone to dying, whereas producing too much of it has a protective effect (Wang et al. 2011). In the R6/2 mouse model of Huntington’s disease, melatonin reduces the rate of disease progression by preventing mitochondrial cell death. In R6/2 mice, a decrease in MT1 but not MT2 melatonin receptors is found in the brain; this reduction can be partially alleviated by administering melatonin (Wang et al. 2011). Research indicates that the creation of selective MT1 receptor agonists may result in neuroprotective medications that can treat individuals afflicted with Huntington’s disease.

Unlike other receptors, the activation of MT2 receptors has been associated with melatonin’s protective effects against neuronal damage in the aftermath of ischemic strokes, according to Mozaffarian et al. (2015). Melatonin treatment also provides protection against ischemia/reperfusion injury in a mouse stroke model by receptor-mediated pathways that are blocked by 4P-PDOT or luzindole. In addition, melatonin stimulates neurogenesis and cell multiplication via a mechanism that relies on the MT2 receptor (Chern et al. 2012). Consequently, these findings indicate that the MT2 receptor plays a part in mediating the neuroprotective effects of melatonin after ischemia/reperfusion and is associated with a significant increase in neurogenesis.

### Mechanisms and pathways of melatonin action

Melatonin demonstrates versatile behavior that affects numerous physiological processes. Many in vitro studies show melatonin’s surprising therapeutic and ameliorative effects on several cancer cell lines and their apoptosis. Concerning the International Union of Basic & Clinical Pharmacology (IUPHAC), different forms of melatonin receptors, either high (MT1) or low affinity (MT2), which interact with intercellular proteins, such as RAR-related orphan receptor (ROR), retinoid Z receptor (RZR), and calmodulin, have been identified. MT1 and MT2 are officially nomenclated as Mel1a and Mel1b. These proteins are assigned to the heterotrimeric GTP-binding protein family and share a similar amino acid sequence (Dubocovich et al. 2010).

The MT1 receptor is 351 amino acids long which is encoded in human chromosome 4. It is found in human skin to a large extent, and initiates adenylate cyclase inhibition by coupling with different G-proteins (Emet et al. 2016). During the course of ageing, and also AD, the expression levels of MT1 receptor in cortex and suprachiasmatic nucleus (SCN) regions of the brain decrease (Wu and Swaab 2007).

On the other hand, the MT2 receptor is composed of 363 amino acids that the coding region is located in the human chromosome 11. Similar to MT1 receptor, MT2 receptor also inhibits adenylate cyclase, resulting in the cessation of cAMP production. In addition, MT2 receptor inhibits the activation mechanism of soluble guanylate cyclase.

Linoleic acid plays a key role in the proliferation of tumor cells. It is mainly used in the biosynthesis of prostaglandin and cell membranes. During cell narcosis, linoleic acid oxidized to 13-hydroxy octadecadienoic acid (13-HODE) in the presence of 15-lipoxygenase, which the product itself serves as an energy source for tumor signaling molecules. Since both MTL1 and MTL2 partly inhibit the signaling through adenyl cyclase and cyclic AMP (cAMP), a consequent decrease in cAMP production results in lower cellular uptake of linoleic acid (Chang et al. 2015). The limitation on linoleic acid uptake by cancer cells is shown to be caused by the active role of melatonin, which tends to launch anti-proliferative strategies (Blask et al. 1999).

Some other studies suggest the existence of a prototype melatonin receptor known as the G-protein coupled receptor 50 (GPCR50); however, its function is obscure. It is presumed that GPCR50 plays a role in hypothalamic functions, especially in the interaction of a regulatory protein with MT1 receptor. With respect to the recent studies involving mass spectroscopy and enzymatic analysis, another enzyme, which is formerly known as quinone reductase 2 (QR2), is associated with melatonin interaction, and renamed to MT3 receptor subsequently (Karunanithi et al. 2014). MT3 receptors are mainly found in muscle, kidney, liver, intestine, heart, and brown tissues. It aids in reducing oxidative stress through the inhibition of electron transport reactions of quinones (Hardeland and Pandi-Perumal 2005). Regarding earlier studies, melatonin can also bind to nuclear hormone receptors, which are associated with retinoic acid (Cook et al. 2015).

The mechanisms involved in the cancer cell inhibition by melatonin are listed as antioxidation, epigenetic alteration, anti-angiogenesis, cell cycle arrest, regulation of estrogenic receptor expression, telomerase reverse transcriptase depletion, apoptosis and differentiation, and changes in energy metabolism (Mediavilla et al. 2010). The free radical scavenging properties and antioxidant activity of the melatonin are crucial in terms of its anti-carcinogenic activity (Galano et al. 2013). Melatonin decreases the expression of the estrogenic receptor and restricts the binding of the estradiol complex to the estrogen response element on DNA (Lopes et al. 2016). The disruption of estrogenic signaling by melatonin knocks the calmodulin out, by-passing its assistance in the initiation of anti-carcinogenic activity (Martínez-Campa et al. 2009). Moreover, melatonin has an inductive effect on telomerase activity that initiates pro-apoptosis impacts on tumor cells (Leon-Blanco et al. 2003). With regards to the study conducted by Guerrero et al., melatonin can alter both specific and non-specific immunity parameters (Guerrero and Reiter 2002). Furthermore, it may regulate cytokine production and act as an immune system enhancer. Lymphoid organs, such as bone marrow, thymus, lymphocytes help melatonin production. Melatonin shows anti-carcinogenic activity also through binding directly to the cell surface receptors or binding nuclear receptors of natural killer cells, leukocytes, monocytes, interleukins (IL-2, IL-6, IL-12), tumor necrosis factor-alpha, and interferon-gamma (Carrillo-Vico et al. 2013).

Nuclear receptors display important structural similarities with retinoid receptors (ROS and RZR) and vitamin D receptor. Recent studies on the cellular effects of melatonin have revealed that melatonin primarily acts on various signal transduction systems, such as inhibition of adenosine phosphorus esters and Ca^2+^ mobilization, inhibition of arachidonic acid release, activation of protein kinase C, protein C inhibition of adenylyl cyclase, and opening of potassium channels (Campbell et al. 2010). The observations of Mediavilla et al. exposed that melatonin downregulates the tumor cell cycle progression through the upregulation of p21/WAF1 and p53 suppressor genes. The same study has presented that the viability of tumor cells is diminished within 48 h after melatonin treatment under physiological conditions (Mediavilla et al. 2010). Another possible anti-carcinogenic mechanism of melatonin is thought to be the inhibition of HIF-1α protein expression, which ends up with the downregulation of vascular endothelial growth factor as a punishment for tumor cells (Lv et al. 2012).

The anticancer effect of melatonin is not only confined to the epithelial and endothelial level, but it also has a bone protective effect, which was proven by a specific study with the finding of maximal MT1-mRNA expression and lower OPG-mRNA levels in osteosarcoma cells (Cutando et al. 2011). In addition to the aforementioned findings, normal human osteoblast cells and bone marrow cell lines exhibited higher OPG-mRNA expression and lower MT1-mRNA levels. These results significantly underline the essential importance of MT1 receptor in the field of bone oncology. In detail, melatonin has been found to downregulate D1, CDK4, cyclin B1, and CDK1 protein expression in a dose- and time-dependent manner in order to prevent the carcinogenic activity of MG-63 osteosarcoma cell line (Casimiro et al. 2012).

Besides the pathways related to receptors, melatonin might also exert anticancer effects through aforementioned complex mechanisms. Melatonin can control the intercellular redox state to produce an anti-proliferation effect. This anti-proliferation effect highly relies on the exhaustion of intercellular reactive oxygen species in addition to the increase of intercellular glutathione and GSH levels. Nevertheless, stimulation of hydrogen peroxide production can hasten cell death. Thus, intercellular redox level escalation enables melatonin to manifest its anti-carcinogenic actions. Another critical factor for differentiation in cancer cell lines is appropriate enzyme activation. In antiblastic therapy and tumor etiopathogenesis, melatonin enhances the Amine Precursor Uptake and Decarboxylation system to channel its anticancer activity (Boyd 2001). Diffuse neuro-endocrine system, which produces biologically active molecules such as serotonin and melatonin, has a crucial role in various initiation and progression steps of proliferation (Kvetnoĭ and Raĭkhlin 1978). The final stages of cancer proceed with the reduction of these molecules, resulting in increased tumor cell proliferation. With that being said, it should not be assumed that the anticancer activity of melatonin is limited to the mechanisms and pathways mentioned above. Yet, further research is necessary to distinguish between the complex mechanisms and selective pathways that are induced by melatonin in anticancer studies.

### Melatonin and cholinergic system

**Fig. 2:**
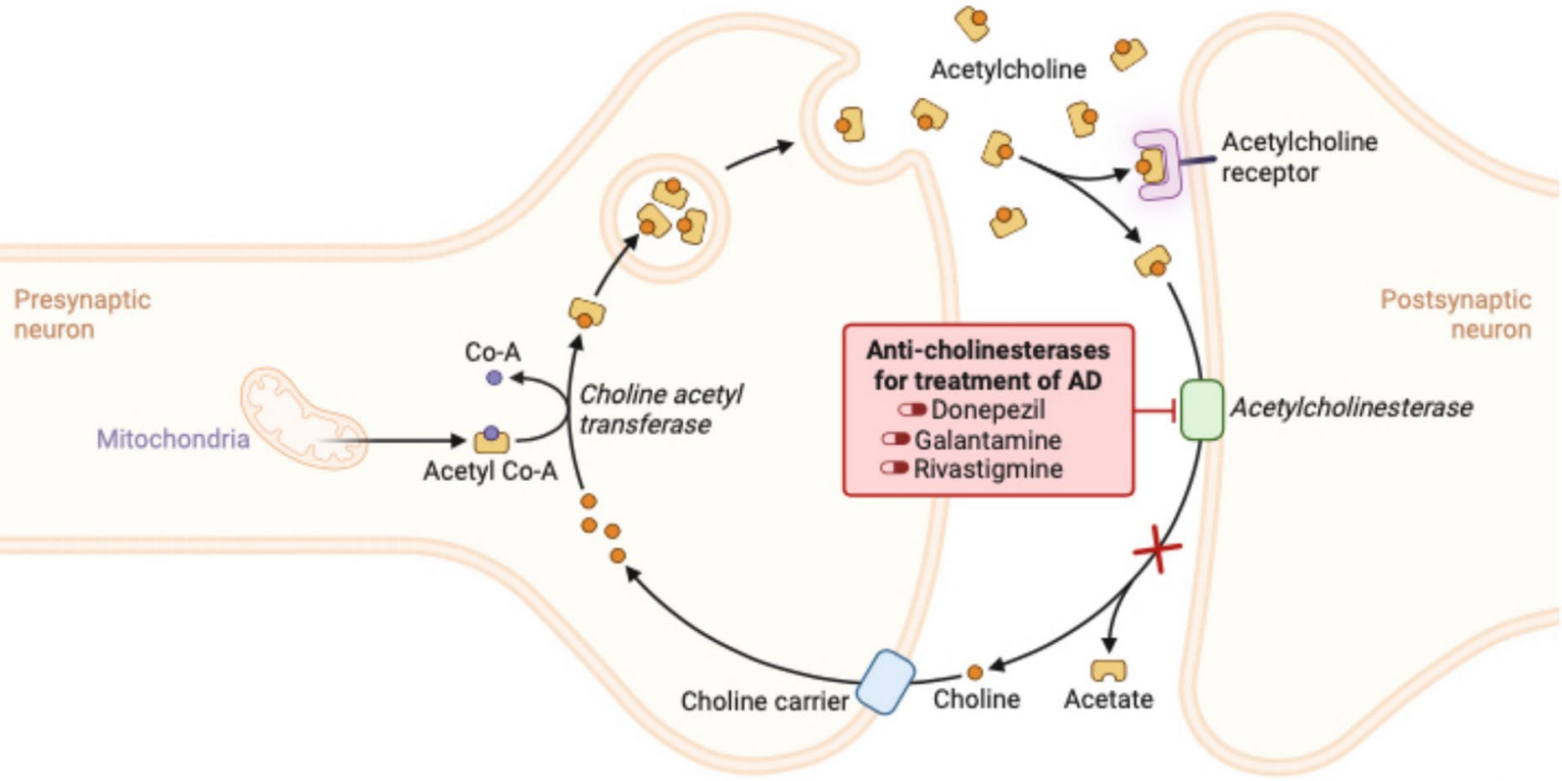
Acetylcholine release and cholinergic transmission. The primary mechanisms of action of widely used drugs in the treatment of Alzheimer’s disease involve the inhibition of acetylcholinesterase, an enzyme responsible for breaking down acetylcholine, as seen with the medications donepezil, rivastigmine, and galantamine. Inhibiting acetylcholinesterase helps increase acetylcholine levels in the brain, potentially enhancing cognitive function in patients with Alzheimer’s disease. Acetylcholine (ACh) functions as a neurotransmitter within both the central nervous system and the parasympathetic nervous system, being synthesised from choline and acetyl CoA by choline acetyl transferase. Acetylcholine is transported into the synaptic cleft via synaptic vesicles and there is either broken down by the enzyme Acetyl cholinesterase (AChE) into its components acetate and choline or it binds to its receptor. Choline is reabsorbed into the axonal terminal for additional acetylcholine synthesis, whereas acetate disperses into the surrounding environment. In the presence of an inhibitor, AChE activity is reduced, resulting in an increased level and prolonged duration of the neurotransmitter’s action. Created with BioRender.com

Recent research has found a connection between cognitive impairments and the malfunctioning of neurotransmitters or their corresponding receptors. Disruptions to the cholinergic system and regulatory issues with various neurotransmitters, including dopamine, norepinephrine, 5-hydroxytryptamine, glutamic acid, and γ-aminobutyric acid, lead to post-operative cognitive dysfunction (POCD) (Zhang et al. 2020). The central cholinergic system oversees and controls advanced cognitive processes of the brain, including memory, learning, dendritic branching, neuronal growth and development, and differentiation. The optimal outcome of anesthesia is contingent upon the diminution of acetylcholine release and the suppression of cholinergic transmission. Many of the drugs employed in anesthesia interact with nicotinic and muscarinic receptors, resulting in significant impacts on brain functions via a chain of synaptic and postsynaptic events (Fodale et al. 2006) (Fig. 2). As we age, the brain’s neuron count, particularly in areas such as the temporal lobe cortex and hippocampus, diminishes; consequently, the loss of synapses leads to a gradual decline in critical functions, including memory and learning. Significant reductions in the number of cholinergic neurons in the brain due to anesthesia and surgical factors worsened the cognitive decline associated with degeneration (Xu et al. 2019a, b). Researchers Ni et al. conducted a study to examine how melatonin affects neurodegenerative damage resulting from anesthesia, finding that isoflurane anesthesia reduces choline acetyltransferase expression in the hippocampus, increases Aß formation, and results in cholinergic system dysfunction. They have also found that a 7-day treatment of 10 mg/kg melatonin prior to anesthesia can prevent the formation of Aß in mouse models, boost ChAT expression, and safeguard the cholinergic system, potentially lessening the likelihood of neurodegenerative disease onset (Ni et al. 2013). Researchers led by Corrales treated a group of mice with Down syndrome with 0.5 mg of melatonin daily for 5 months. As a result, they found that cholinergic degeneration was reduced, and the mice showed improvements in both memory and spatial learning (Corrales et al. 2013). Labban et al. conducted a study in which AD mice models were treated daily with 80 mg/kg melatonin over the course of 8 weeks. The outcomes of this study indicated that melatonin increased acetylcholinesterase levels in addition to facilitating BDNF/CREB1 protein expression in the prefrontal cortex of mice, and it also led to improvements in memory and passive avoidance performance, as reported by Labban et al. in 2021. Researchers led by Chen et al. discovered that administering scopolamine intraperitoneally to mice resulted in a substantial reduction of immunoreactivity and protein levels of the ChAT enzyme, high-affinity choline transporter, vesicle acetylcholine transporter, and MT1 receptor in the brain’s septum and hippocampus, ultimately impairing spatial learning and memory. Melatonin treatment successfully reversed these outcomes and has the potential to be a therapeutic option. Research has provided insight into the beneficial impact of melatonin on cognitive impairments in multiple disease models by restoring the function of the cholinergic system.

The association between beta-amyloid (Aβ) and the cholinergic system suggests that alteration in acetylcholine levels may reverse the inhibition of long-term potentiation by Aβ. Long-term amplification, a phenomenon in which response amplitude increases with the application of high stimulation frequencies in a neuronal circuit, is a possible mechanism of memory and a learning mechanism. Therefore, acetylcholine augmentation could be considered as a main therapeutic agent in AD (Lin et al. 2013). Another study revealed that melatonin prevents peroxy-nitrite from blocking the transfer of acetylcholine from synaptic vesicles. Aβ has a major role in stimulating glutamate release by microglial cells and preventing neurons from accumulating excess glutamate, which means that Aß basically blocks glutamate uptake. In another study, administration of kinetic acid led to glutamate release via N-methyl-D-aspartate (NMDA) receptor and caused brain damage. They have observed that glutamate receptor activation activated NOS, leading to an increased Ca^2+^ influx through NMDA-controlled channels via NO synthesis (Besancon et al. 2008). It has been clarified that the effect of abnormal glutamate secretion is due to the toxic impact of enhanced glutamate levels on neurons through NMDA receptors in AD. Both glutamate receptor degradation and an increase in glutamate secretion levels can cause this abnormality. In contrast, melatonin reduces glutamate synthesis and NMDA induction. It has been revealed that apoptosis induced by glutamate hypersecretion in hippocampal regions of the brain is reduced by melatonin administration (Wang et al. 2019a, b). Besides melatonin, hippocampal MT2 receptors may prevent the progression of learning deficits in mice models. Several studies with mice models have shown that melatonin introduction reduces glutamate levels and diminishes the incidences of structural defects caused by hypoxia induction in axons and dendrites of neurons (Jürgenson et al. 2019).

Initial research on the human brain concentrated on the suppression of acetylcholinesterase (AChE), one of two cholinesterases that break down acetylcholine, along with butyrylcholinesterase (BuChE), as a therapeutic method for dementia that utilises a cholinergic approach. The increasing recognition of BuChE’s role is evident in both healthy and diseased brains. Research indicates that levels of the enzyme BuChE increase and may assume the role of metabolizing acetylcholine at synapses as the activity of AChE declines due to the gradual loss of cortical neurons in Alzheimer’s disease (Greig et al. 2005; Mesulam et al. 2002). Additionally, studies have found that the rate of cognitive deterioration in dementia with Lewy bodies is associated with BuChE levels in the temporal cortex (Perry et al. 2003). In comparison, a dual inhibitor of AChE and BuChE may offer at least a theoretical advantage over a selective AChE inhibitor.

Rivastigmine, classified as a carbamate-type dual inhibitor of AChE and BuChE, is commonly utilised as a cholinergic agent in the symptomatic management of Alzheimer’s disease; its efficacy and tolerability in this condition were reviewed in a previous study (Spencer and Noble 1998). Rivastigmine selectively blocks AChE and BuChE in the brain, thereby increasing acetylcholine levels by making it more available in synapses (Spencer and Noble 1998). The cholinergic deficiency associated with Parkinson’s disease-related dementia is thought to be addressed by the drug in a way that enhances cholinergic activity (Bohnen et al. 2003). The drug facilitates proper acetylcholine transmission in areas such as the hippocampus and cortex by lowering the degradation levels of acetylcholine released by intact cholinergic neurons (Spencer and Noble 1998). Following oral administration of 1–6 mg rivastigmine twice daily, AChE activity is suppressed in a dose-dependent fashion within the CSF of individuals with Alzheimer’s disease (Cutler et al. 1998). Rivastigmine suppresses AChE and BuChE activities in CSF to a similar degree in patient populations. Research has shown that patients who use rivastigmine for 12 months exhibit a sustained inhibition of both cholinesterases, as demonstrated by Darreh-Shori et al. (2002). Following 12 weeks of treatment with rivastigmine in dosage ranges of 3–12 mg/day, a total of 19 patients with Parkinson’s disease and dementia showed a significant increase in the relative amplitude of alpha waves in their quantitative EEG results, which likely signified enhanced arousal or an improvement in their cognitive condition (Fogelson et al. 2003). Studies by Ceravolo et al. (2006) found that cholinesterase inhibitors, such as rivastigmine at a daily dosage of 6–12 mg, enhanced regional cerebral blood flow in individuals with Parkinson’s disease dementia. In 17 patients, a highly significant increase in blood flow in both frontal and parieto-temporal areas of the brain was noted after six months of treatment using a cholinesterase inhibitor, with the increase being highly statistically significant at p < 0.001, as reported by Ceravolo et al. in 2006.

Rivastigmine is approved for the treatment of mild to severe Alzheimer’s disease-related dementia in both Europe and the United States. In a well-designed trial, rivastigmine was generally well accepted and notably reduced cognitive and functional decline in individuals with Parkinson’s disease dementia. Recent American Academy of Neurology guidelines recommend Rivastigmine as a treatment option for alleviating symptoms in patients with Parkinson’s disease dementia, as reported by Miyasaki et al. (2006).

### Ageing

Approximately 30 years ago, the initial long-lived species was isolated from C. elegans, marking the beginning of a new era in aging research as described by Klass (1983). Research conducted in 1925 discovered that the light intensity can impact the growth rate and lifespan of Drosophila, as found by Northrop (1925). Furthermore, research has shown that caloric restriction impacts the ageing process, age-related health issues, and lifespan in mice and rats, as reported by McCay et al. (1975). Research on the subject has been generating significant interest, with a strong indication that the flexibility of the ageing process plays a key role in extending lifespan. About 30 years ago, the first long-lived species was isolated from C. elegans and this discovery ushered in a new era of ageing research (Klass 1983). For the past ten years, research has indicated a possible significant connection between the process of ageing and many chronic health conditions in humans. This connection has been observed in diabetes (Wilkerson 1947), Alzheimer’s disease (Cortes-Canteli and Iadecola 2020), Parkinson’s disease (Hoehn and Yahr 1967), cardiovascular diseases (Kaufman and Poliakoff 1950; Yan et al. 2021), chronic obstructive pulmonary disease (Hernández Cordero et al. 2022), osteoporosis (Grunewald et al. 2021), and even osteoarthritis (Chen et al. 2022). Patients over 60 often suffer from a multitude of health conditions as they grow older. Patients with these conditions require a combination of various treatments in order to achieve therapeutic benefits over the long term (Ding et al. 2023). Furthermore, the various treatment options available for age-related diseases often interact with one another (Bettonte et al. 2022). Understanding the ageing process is crucial for identifying therapeutic targets for age-related diseases and developing pharmacological agents that could be used in future clinical trials.

#### The role of melatonin in ageing

Ageing is a multifactorial process that results in degeneration and dysfunction at the genetic, cellular and organismal level, leading to the decline of maintenance mechanisms and the exponential accumulation of molecular damage. Senescent organisms exhibit genetically significantly reduced adaptive potential and are unable to overcome diverse stress factors and adverse external stimuli (Poeggeler et al. 1993). Ageing refers to post-maturational changes that underlie increased vulnerability to adversity; therefore, reducing the ability to survive. As age advances, and age-related diseases manifest or progress, the biosynthesis of melatonin, predominantly nocturnal, is significantly reduced in various species, including humans (Reiter 1992). Delaying the rate of aging and the onset of age-related diseases can be achieved through melatonin supplementation or therapeutic methods that sustain the high amplitude of the body’s natural melatonin production cycle (Poeggeler et al. 1993). Melatonin’s natural daily cycle amplitude diminishes in older adults and is nearly absent in certain neurodegenerative disorders, including Alzheimer’s disease (Wu and Swaab 2005). A disrupted melatonin circadian rhythm could have significant effects on the health and wellbeing of elderly individuals. Healthy young organisms display a significant circadian rhythmicity in various crucial physiological processes, including the sleep–wake cycle, core body temperature, performance, wakefulness, and the secretion of numerous hormones. These rhythms can have a significant impact on maintaining health and overall well-being. Old age is typically marked by the disturbance of the regular circadian patterns of these cycles, with decreased peak values, timing difficulties, disorganization of the temporal sequence, and impaired response to environmental time cues (Karasek 2004). Melatonin functions as a timekeeper signal, known as a Zeitgeber, according to Armstrong and Redman (1991).

Research indicates that periodic melatonin administration can reset internal rhythms, synchronizing them and increasing their amplitude to normal levels in aged organisms (Armstrong and Redman 1991). A number of theories have been put forward suggesting a connection between the pineal gland and melatonin and the mechanisms of ageing and its associated conditions. The decrease in circulating melatonin levels over the course of a person’s lifetime, which coincides with a more general decline in many circadian rhythms as people get older, is a compelling indicator that melatonin, even at physiological levels, may have an important role to play in this process.

These geronto-protective effects can combine to result in a substantial improvement in well-being, as well as a decrease in the occurrence and severity of specific age-related health issues characteristic of the elderly (Reiter et al. 2002b). Research on animals has shown that low levels of melatonin, when taken over a prolonged period, may counteract the ageing process by reversing and stabilizing the negative effects it has on oxygen and energy metabolism (Okatani et al. 2003).

The electron transport chain inside mitochondria ensures a secure four-valent reduction of molecular oxygen to form water. The decrease in molecular oxygen’s valency due to electron loss leads to the formation of ROS (Watabe et al. 2007). Aging cells exhibit elevated electron leakage rates due to compromised electron transfer chain function. High levels of ROS can lead to an overactive response in redox signaling pathways, ultimately increasing inflammation, cancer, cell death, and contributing to accelerated aging, as noted in Schieber and Chandel’s 2014 study. Melatonin minimizes electron loss in mitochondria and acts as an antioxidant, providing a high electron supply to neutralize ^1^O_2_, O^2^∙-, O_2_^2−^, and OH∙ radicals through reactions that do not follow a one-to-one ratio (Tan et al. 2000).Melatonin and its derivatives exhibit a reductase-modulatory effect on ROS and reactive nitrogen species (RNS) (Wang et al. 2019b). Melatonin-induced redox regulation may be achieved through direct ROS scavenging and the action of both enzymatic and non-enzymatic antioxidant systems, as described by He et al. (2017). Exogenous non-enzymatic antioxidants, which are derived from nutritional sources, include vitamins A, E, C, xanthophylls, polyphenols, and carotenoids, as noted by Okeke et al. (2022). Vitamin C functions as a hydrophilic antioxidant, whereas other compounds like vitamins A and E, polyphenols, and carotenoids are active in a hydrophobic environment, as reported by Kuciel-Lewandowska et al. (2020). The direct antioxidant properties of melatonin are dependent on its own electron-rich aromatic indole ring. Indole ring makes it a potent electron donor that can significantly reduce free radicals (Mannino et al. 2021). Melatonin neutralizes reactive oxygen species (ROS) and free radicals by donating electrons to them. This process converts melatonin into its radical form, N-acetyl-5-methoxytryptamine (AMT). The radical form is relatively stable and can react with additional ROS, leading to the formation of less harmful products. The oxidized form can be restored to its active state through enzymatic reactions. The primary enzyme involved in this regeneration is glutathione peroxidase, which reduces oxidized melatonin using reduced glutathione (GSH). The metabolism of the substance yields various metabolites, including 6-hydroxymelatonin, which exhibits antioxidant properties and plays a role in the body’s overall antioxidant protection mechanism. In essence, melatonin’s redox cycling involves its capacity to donate electrons to counteract oxidative stress, produce stable radicals, and be restored by antioxidant enzymes, particularly glutathione peroxidase, thereby enabling it to function effectively as an antioxidant. The enzyme glutathione peroxidase (Sies and Jones 2020) is responsible for maintaining melatonin within the oxidoreduction redox cycle. This enzyme is crucial for the replenishment of melatonin by converting its oxidized form back into its active state with the aid of reduced glutathione (GSH) as a co-factor. Glutathione peroxidase functions to facilitate the reduction of hydrogen peroxide (H₂O₂) and other peroxides through the utilization of GSH. During this process, GSH is converted into glutathione disulfide (GSSG). When melatonin donates electrons, it is converted into its oxidized form, helping to neutralise free radicals. Glutathione peroxidase enables the transfer of electrons from GSH to the oxidised form of melatonin, thereby restoring its active form. Maintaining redox balance in cells relies on this enzymatic action, enabling melatonin to act as a potent antioxidant that counteracts oxidative stress (Hasan et al. 2018; Rossi et al. 2023).

Melatonin is oxidized when it provides electrons to ROS or RNS (Hardeland 2021). Typically, this process involves either the loss of hydrogen atoms or the addition of oxygen atoms to the melatonin molecule. The main oxidised form of melatonin is N-acetyl-5-methoxytryptamine (AMT), alongside other metabolites including 6-hydroxymelatonin. The oxidized forms of melatonin exhibit distinct chemical compositions when contrasted with the original melatonin molecule. Melatonin’s molecular configuration and functional groups are modified by oxidation, thereby impacting its reactivity, solubility, and biological effects. The introduction of hydroxyl groups to oxidized forms enhances their capacity for interaction with other molecules (Galano et al. 2011). Melatonin’s electron donation leads to structural changes, resulting in the formation of different oxidized metabolites that may retain some of the antioxidant properties, but are chemically distinct from the original hormone (Hardeland 2017).

Mitochondrial function decreases with age, with a notable impact on the activity of complexes I and III. These complexes are essential for the electron transport chain and ATP production, as noted by Miwa et al. (2022). The buildup of reactive oxygen species (ROS) can result in damage to mitochondrial DNA, proteins, and lipids, as found by Radak et al. (2011). Oxidative damage interferes with the operation of complexes I and III, thereby diminishing their capability for electron transfer (Choksi et al. 2007). Mitochondrial DNA mutations can accumulate over time, resulting in dysfunctional proteins that are essential components of the electron transport chain. Electron flow is consequently reduced and ATP synthesis is hindered as a result. The composition of mitochondrial membranes undergoes changes as people age, which impacts the fluidity and operational efficiency of electron transport chain complexes. The change in question hinders the correct formation and functioning of complexes I and III, as noted by Genova and Lenaz (2015; Gómez and Hagen 2012). Coenzyme Q10 (ubiquinone) plays a crucial role in facilitating electron transfer between complexes I and III. Reduced coenzyme Q10 levels in aging individuals can hinder electron flow and bioenergetic processes, as reported in the studies of Ebadi et al. (2001) and Banerjee et al. (2022). This can ultimately contribute to the destabilization of mitochondrial complexes I and III due to the combined effects of oxidative stress, genetic mutations, altered lipid composition, decreased coenzyme Q10 levels, and impaired mitochondrial dynamics. A disruption in electron flow ultimately compromises essential bioenergetic processes that are necessary for cellular function and contributes to the aging phenotype.

Research has demonstrated that melatonin affects the patterns of neurodegenerative genes and proteins, especially in cases of age-related disruptions to our internal body clock. The significance of this effect lies in the fact that disruptions to circadian rhythms can worsen neurodegenerative processes. The regulation of circadian rhythms is dependent on interconnected feedback systems that balance gene expression through continuous interaction and are comprised of transcriptional and translational elements. Alzheimer’s disease, the leading cause of dementia and a particularly severe psychiatric disorder (Barnes and Yaffe 2011), is distinguished by the formation of amyloid-β (Aβ) deposits outside cells, neurofibrillary tangles (NFTs) composed of abnormally phosphorylated tau, cell loss, and inflammation in the nervous system (Ballard et al. 2011). In addition to significant cognitive impairments, individuals with Alzheimer’s disease experience extreme disruptions to their body’s natural sleep–wake cycles and other related processes. Over the past few years, significant strides have been taken to gain a deeper understanding of the molecular and cellular causes of sleep disturbances and disrupted circadian rhythms that are commonly seen in AD patients (Bliwise et al. 2011). The clock pathway plays a vital role in maintaining rhythmicity, and research connecting this molecular group to brain and body functions in AD patients may help clarify the underlying causes of this prevalent form of dementia (Harper et al. 2004). A network of extra brain regions outside the SCN contains self-sustaining oscillators that facilitate circadian function, as noted in Cermakian and Boivin (2009). These secondary oscillators, similar to the SCN, depend on feedback loops that include clock genes and proteins.Research suggests that the function of the clock pathway in the suprachiasmatic nucleus (SCN) and other brain areas is thought to be distinct and related to the specific function of each region (Cermakian and Boivin 2009). Studies in rodents have found that age-related decline in circadian rhythm regulation occurs at the cellular level within the SCN, and is associated with altered expression of multiple genes crucial for circadian clock function (Asai et al. 2001). Additionally, it has been discovered that light-induced expression of clock genes is modified in the SCN of aging hamsters (Kolker et al. 2003), and that aging affects phase-shifts of the day-night cycle in the pineal gland and arcuate nucleus of aged rats (Davidson et al. 2008). Targeted deletions of Clock or Bmal1 genes significantly impact the rate of ageing by disrupting the circadian system, resulting in increased brain inflammation and neurodegeneration (Dubrovsky et al. 2010). In addition to regulating the circadian rhythm, clock proteins could have other roles (Kondratova and Kondratov 2012). Evidence from mouse models demonstrates accelerated neurodegeneration in mice with brain-selective or conditional Bmal1 knockouts, as observed in studies by Yang et al. (2016). It is therefore plausible that the upregulation of Bmal1 and the modified activation of the negative feedback loop, which takes place in all brain areas, acts as a counterbalancing mechanism to the neurodegenerative effect. Further investigation into these aspects is required, and this can be achieved using various circadian mutant models. Altered expression of the clock gene causes a loss of timely coordination among different brain areas, interferes with the regulation of basic cellular metabolic and homeostatic processes, and ultimately results in the disruption of connections among brain structures, ultimately leading to cognitive impairment and the characteristic chrono-disruption seen at the onset of early neurodegenerative diseases in patients.

#### Aging and autophagosome biogenesis

Autophagosome formation rates may fluctuate with age, despite autophagy-related protein levels remaining stable in neurons over time. Phosphorylation and other post-translational modifications play a substantial part in the autophagy process. Protein modification changes can affect the rate of autophagosome formation without impacting the overall levels of protein production. The formation speed of autophagosomes can also be influenced by the cellular components’ location within the cell. In Caenorhabditis elegans, autophagy activity generally decreases as age advances. Age seems to influence neuronal autophagy to a greater degree of variability than it does other tissues (Chang et al. 2017). Modulating autophagy in neurons can have an impact on longevity and lifespan. In Drosophila, flies lacking the Atg8a gene exhibited shorter lifespans and a higher number of ubiquitinated protein aggregates in their neurons. Overexpressing dAtg8 in the CNS via the APPL-Gal4 driver resulted in the prevention of protein aggregate accumulation and a significant increase in lifespan. Overexpressing dAtg8 pan-neuronally with the aid of ELAV-Gal4, which drives earlier expression, did not lead to an increase in lifespan, indicating that the timing of autophagy induction is crucial (Simonsen et al. 2008). In the nematode C. elegans, blocking components of the initiation and nucleation complexes in neurons post-reproduction led to extended lifespan, whereas blocking these components in pre-reproductive animals resulted in decreased lifespan. Research data indicate that inhibiting the formation of partially completed autophagosomes is beneficial in post-reproductive animals (Wilhelm et al. 2017).

Neurodegenerative disorders linked to aging—specifically Alzheimer’s disease, Parkinson’s disease, Huntington’s disease, Amyotrophic lateral sclerosis, and frontotemporal dementia—are all marked by the accumulation of misfolded proteins and dysfunctional mitochondria (Chu 2019). Data from both experimental models and post-mortem analysis of human tissues indicate that autophagy dysfunction may be a widespread factor contributing to the disease process in these conditions (Boland et al. 2018). Observations of neurodevelopmental or neurodegenerative disease in humans, triggered by mutations in various proteins involved in autophagy, provide additional support for this hypothesis, including those affecting PINK1, Parkin, OPTN, TBK1, ATL1, SQSTM1, and WDR81, as well as ATG5, AP4, HTT, WIPI4, and DYNC1H1 (Zhu et al. 2019). No consensus has been reached regarding which step or steps might be disrupted in major neurodegenerative diseases like Alzheimer’s disease, Parkinson’s disease, or amyotrophic lateral sclerosis. To date, research has shown age-related or disease-linked impairments at various stages of the pathway, encompassing autophagosome formation (De Pace et al. 2018), cargo loading (Rudnick et al. 2017), intracellular transport (Nicolas et al. 2018), and autophagosome-lysosome fusion or acidification (Lie and Nixon 2019), across different disease models. The ubiquitin–proteasome pathway and lysosomal degradation through chaperone-mediated autophagy are among the pathways whose deficits may contribute to neurodegenerative disease (Scrivo et al. 2018). Moreover, it is unclear whether defects in neuronal autophagy are the primary cause or whether age-related neurodegeneration is also influenced by deficits in autophagy or related degradative pathways in supporting cells such as glia. Studies on autophagy and various neurodegenerative diseases are summarized in Table 1.

**Table 1:** Autophagy and various neurodegenerative diseases (NDDs)

NDDs	Autophagy Sites Associated with NDDs	Mechanism of Autophagy in NDDs	Changes in Autophagy	References
AD	PICALM decreases or disappears	Autophagosomes formation, and autophagosome-lysosomal-fusion protein	Reduced autophagosomes degradation	Ando et al. 2020; Van Acker et al. 2019
AD	Beclin1 decreases	Defects in autophagosome synthesis	Reduced autophagosomes degradation	Roy et al. 2014; Wani et al. 2019
AD	PS1 and PS2 mutations	Impaired glycosylation Lysosomes damaged by acidification	Reduced autophagosomes degradation	Frake et al. 2015
HD	Beclin1	Beclin1 level decreased	Reduced autophagosome formation and impaired autophagy	Mealer et al. 2014
ALS	Gene mutation	Interact with LC3	Increased autophagosome and autophagic matrix binding	Kochergin et al. 2019

#### Theories linking melatonin to the ageing process

In mammals, the biogenic amine melatonin is synthesized from the aromatic amino acid L-tryptophane in many tissues; however, circulating levels are mainly of pineal origin with a pronounced nocturnal peak (Reiter 1980a, 1992). With respect to age-related diseases, such as AD, and advancing age, it was indicated that the melatonin concentrations in individuals reduce, which is highly correlated with disease progression and genotype (Reiter 1992; Wu and Swaab 2005).

Research conducted by numerous teams has revealed a decline in melatonin synthesis and release in older animals and humans along with a significant disturbance in the natural circadian cycle of circulating melatonin, resulting in an almost total disappearance of the typical nocturnal peak in melatonin levels, a phenomenon observed in many devastating age-related degenerative diseases. The physiological effects of an ageing pineal gland and its weakened capacity to produce melatonin with age could lead to melatonin deficiency in ageing animals, posing a severe threat to the old organism’s well-being.

Extensive research has been conducted on the decrease in melatonin levels that occurs during both normal ageing and pathological ageing processes in the pineal gland, the brain, and numerous other tissues outside of the pineal gland, as well as in bodily fluids such as cerebrospinal fluid, plasma, saliva, and urine (Wu and Swaab 2005; Reiter 1992). Almost all of the studies reported a highly significant drop in melatonin levels and the production of its primary liver metabolite 6-hydroxymelatonin; with a few exceptions, no statistically significant differences were shown, primarily due to methodological issues stemming from the significant variability of nocturnal melatonin production and 6-hydroxymelatonin excretion, as well as the timing of their age-related decline (Wu and Swaab 2005).

Recent research, which demonstrates the fact that preserving the circadian structure of melatonin production can serve as a significant indicator of biological age and has tremendous potential to use in assessing human health status, is of considerable interest and significance in the context of the present overview. The first reports based on observations that melatonin given to mice with potable water prolonged their lifespan and kept the organism in a younger state were initiated due to the strong immunomodulatory effects of indoleamine (Pierpaoli et al. 1991). Researchers who initially published their findings in the late 1980s and early 1990s have since revised and expanded their theory to propose that the pineal gland and its secreted substances, which may include melatonin and possibly other compounds, serve as an internal control and regulatory mechanism for “self-control”. Their theory posits that ageing is a direct outcome of the loss of "self-control", ultimately leading to the failure of the immune system. It is thought that melatonin and peptides originating from the pineal gland are crucial in controlling the immune system.

Immunosenescence is typically linked to autoimmune diseases and inflammation, and it can significantly speed up the aging process. Experimental data available thus far suggest that melatonin and other pineal products with potential secretory functions play a significant role in immune regulation in rodents (Pierpaoli et al. 1991). Low melatonin levels in the blood may severely compromise this role and put the organism at risk of developing an autoimmune or inflammatory disorder. Melatonin and indole metabolites could potentially be highly effective anti-inflammatory substances. Melatonin and its oxidative byproducts may significantly contribute to its anti-ageing effects, particularly in mitigating the inflammatory conditions commonly found in older adults.

#### Preclinical and Clinical Applications of Melatonin in Neurodegenerative Diseases

Generally accepted clinical research has shown that melatonin and its derivatives have a well-documented safety profile and are associated with minimal side effects when used as supplements or therapeutic agents for short-term periods (Bonomini et al. 2018). Individual responses may differ significantly, and the available long-term safety data is currently restricted, thereby necessitating a cautious approach and further research for distinct populations or particular conditions (Besag and Vasey 2022).

Melatonin is commonly employed for a range of short-term purposes, including the treatment of sleep disorders, managing jet lag, and addressing circadian rhythm irregularities. Studies have demonstrated that it is effective in enhancing sleep quality and shortening the time it takes to fall asleep (Zisapel 2018). Most research suggests that melatonin is generally easy to tolerate, with mild and uncommon side effects reported in the majority of studies. Side effects commonly reported include drowsiness, dizziness, and headaches, as documented by Givler et al. (2023). Research suggests that doses as high as 12 mg or more are typically safe for brief use, provided that individual thresholds for tolerance are taken into account (Besag and Vasey 2022). Long-term ingestion of melatonin is considered safe in the short term, but the long-term effects of taking melatonin remain unclear. Research indicates possible hormonal impacts, especially among youngsters and teenagers, necessitating vigilance (Libowitz and Nurmi 2021). Some studies suggest potential hormonal effects, particularly in children and adolescents, which warrant caution (Libowitz and Nurmi 2021). Safety of melatonin in pregnant and nursing women is not thoroughly documented, and usage should be approached with caution, as noted in studies by Andersen et al. (2016) and Kennaway (2022). Older adults may be more sensitive to melatonin and may need their dosages altered (Anghel et al. 2022). Melatonin may react with a variety of drugs, including anticoagulants, immunosuppressants, and medications impacting the central nervous system (Laurindo et al. 2025). It is crucial to seek advice from a healthcare professional prior to commencing melatonin, particularly if other medications are being concurrently administered.

Evidence from laboratory and animal-based studies that were part of the research showed melatonin has potential for protecting the nervous system. Melatonin therapy has been demonstrated to improve Aβ aggregation, reduce tau hyperphosphorylation, alleviate oxidative stress, decrease neuroinflammation, inhibit neuronal apoptosis, and enhance neuroplasticity impairments. The success of melatonin treatment in animal studies relied on the extent of the disease and the length of the treatment periods. Melatonin administration in young Tg2576 mice inhibited amyloid accumulation and decreased levels of the oxidative stress indicator, nitrotyrosine (Matsubara et al. 2003), whereas no such effects were seen in older Tg2576 mice (Quinn et al. 2005). The efficacy of melatonin appears to be influenced by the severity of Alzheimer’s disease. Additionally, research has indicated that melatonin may be relatively ineffective, potentially due to treatment periods being either too brief or too prolonged. Behavioral improvements were not seen in Aβ-induced Wistar rat models treated with melatonin for a brief period of 10 days, as reported in Eslamizade et al. (2016), and neither were they observed in aluminum-treated Tg2576 mouse models given melatonin for a period of six months, as found in García et al. (2009). Results from animal studies showed a correlation between the ineffectiveness of melatonin therapy and disease severity and treatment duration, which is consistent with findings in patients with Alzheimer’s disease. Melatonin treatment did not demonstrate any advantages in alleviating cognitive impairments and psychiatric and behavioral problems in people with AD at all stages of the condition, although sleep disturbance was only somewhat alleviated in individuals with mild to moderate AD. Additionally, neither short-term nor long-term melatonin therapy was effective in improving symptoms in AD patients. In contrast, melatonin therapy demonstrated beneficial outcomes for sleep, cognitive function, depression, and behavioral symptoms in individuals with mild cognitive impairment. Despite melatonin treatment generally being ineffective in some patients, it remains worthy of further investigation due to its distinct functions in regulating circadian rhythms and mitigating oxidative stress. It is widely acknowledged that melatonin levels decline with typical aging, and that the risk of neurodegenerative diseases escalates with advancing age (Roy et al. 2022). It is thought that a low level of melatonin could be a significant risk factor for the advancement of Alzheimer’s disease as stated in Lahiri et al. (2004). Melatonin treatment may enhance patients’ circadian rhythms and sleep quality, and also lower the likelihood of disease progression. Melatonin’s antioxidant properties differ significantly from antioxidant supplements like vitamins C and E. Antioxidant supplements could produce pro-oxidant by-products as they neutralize free radicals, potentially offsetting the benefits of antioxidants in preventing aging and disease, resulting in antioxidant insufficiency and increased mortality rates (Bjelakovic et al. 2007). Melatonin does not produce pro-oxidant intermediates when neutralizing free radicals (Poeggeler et al. 2010), suggesting its antioxidant activity is even more effective. Further research on this beneficial property is warranted due to its possible relevance in neurodegenerative diseases.

Research into melatonin has been conducted to explore its impact on the clinical manifestations of neurodegenerative disorders, largely due to its demonstrated benefits in various experimental cell and animal studies. The initial case study involved a pair of twins afflicted with AD and demonstrated that melatonin therapy resulted in improved cognitive dysfunction, behavioral problems, sundown syndrome, and sleep quality during the treatment period (Brusco et al. 1998). A condition known as sundown syndrome or sundowning, characterised by its occurrence in the late afternoon or early evening, is typically found in patients with Alzheimer’s disease (Khachiyants et al. 2011). A case study of a man experiencing typical sundown syndrome found that taking a dose of 2 mg of melatonin at 8 p.m. nightly for one week yielded improvements in sleep and behavior, and further gradual improvements were observed when an additional 2 mg dose was taken at 3 p.m. for two weeks, as documented by Lammers and Ahmed (2013). The incidence of rapid eye movement sleep behavior is also decreased by melatonin, as found in the study by Anderson et al. (2008). Other research indicates that melatonin may not alleviate symptoms for all individuals with Alzheimer’s disease. Evidence suggests that melatonin can enhance a patient’s natural sleep–wake cycle, decrease daytime drowsiness, and promote a more positive mood in at least one case. While melatonin had a positive impact on cognitive impairment in one patient, it did not alleviate other symptoms in that individual. Various clinical studies (Cardinali 2019; Pandi-Perumal et al. 2013) have yielded evidence supporting the benefits of melatonin in addressing sleep issues and cognitive decline. Furthermore, research has found that combining exposure to bright light and taking melatonin supplements can enhance sleep–wake rhythms, reduce sundowning symptoms and improve sleep quality in people with Alzheimer’s disease (Uddin et al. 2020). Melatonin, which has been found to stabilise circadian rhythms, diminish daytime drowsiness, enhance sleep quality and slow the advancement of cognitive decline in the majority of case reports and clinical trials, does not yield noticeable benefits for some AD patients in certain research studies. Additional research is required to verify the effectiveness of melatonin for treating clinical symptoms in patients with Alzheimer’s disease. Adjuvant therapies that can be used in conjunction with melatonin supplementation are also worthy of consideration. Studies have found differences in the natural levels of melatonin in patients with PD who are taking melatonin supplements (Kakhaki et al. 2020). Clinical investigations have been conducted to examine the effects of external melatonin on the symptoms exhibited by patients suffering from Parkinson’s disease. Two double-blind and placebo-controlled clinical trials demonstrated an improvement in sleep disruptions among patients with Parkinson’s disease following melatonin therapy, as documented by Sugumaran et al. (2024). A limited number of clinical studies were conducted to examine the effectiveness of melatonin in other neurodegenerative conditions, including ALS and MS. The initial melatonin treatment for ALS took place in three participants who received 30–60 mg of slow-release melatonin orally each night for 13 months. Studies have demonstrated that a patient with advanced ALS experienced a slowed progression of the disease after receiving melatonin, while two other patients exhibited decreased rates of decline at a subsequent evaluation, as reported by Jacob et al. (2002). A clinical trial was conducted among 31 ALS patients who were given 30 mg of melatonin daily, at bedtime, for a period of 24 months as part of their adjunctive treatment. Researchers found that a high dose of melatonin could be used in clinical trials to decrease oxidative stress in ALS patients, but it does not appear to have a neuroprotective impact on the symptoms of the disease (Chen et al. 2020a). In addition, a number of clinical trials investigating the effectiveness and safety of administering melatonin to people with MS have been conducted by Skarlis and Anagnostouli (2020).

## Conclusion

Overall, melatonin appears to be a promising therapeutic agent in the battle against neurodegenerative diseases commonly found in older adults. The compound’s complex mechanisms, encompassing strong antioxidant capabilities, anti-inflammatory actions, and circadian rhythm regulation, establish it as a vital component in safeguarding brain health. Melatonin’s capacity to remove free radicals and decrease oxidative stress holds particular importance, given that oxidative damage is a defining characteristic of neurodegenerative disorders. Melatonin’s ability to control neuroinflammation by suppressing pro-inflammatory cytokines and promoting anti-inflammatory pathways highlights its potential to reduce the neuroinflammatory processes that lead to neuronal degeneration. Restoring circadian rhythms can also enhance the quality of sleep and overall cognitive function, which are frequently disrupted in older populations. Further investigation is necessary to thoroughly understand melatonin’s effectiveness and safety in medical environments. Comprehensive, large-scale clinical trials are required to establish the best dosage levels, treatment periods, and potential interactions with commonly used medications among the elderly population. As research into the aging process and neurodegenerative diseases continues to advance, melatonin is likely to play a crucial part in creating effective plans to improve brain health and the overall quality of life for older individuals. Melatonin serves as a promising solution in the fight against neurodegenerative disorders, providing a natural and potentially effective means of maintaining cognitive abilities and supporting a healthy aging process.

## Data Availability

No datasets were generated or analysed during the current study.

## References

[CR1] Abo Taleb HA, Alghamdi BS (2020) Neuroprotective effects of melatonin during demyelination and remyelination stages in a mouse model of multiple sclerosis. J Mol Neurosci 70(3):386–402. 10.1007/s12031-019-01425-6 31713152 10.1007/s12031-019-01425-6

[CR2] Abraham WC, Jones OD, Glanzman DL (2019) Is plasticity of synapses the mechanism of long-term memory storage? NPJ Sci Learn 4(1):9 31285847 10.1038/s41539-019-0048-y PMC6606636

[CR3] Adamah-Biassi EB, Zhang Y, Jung H, Vissapragada S, Miller RJ, Dubocovich M (2014) Distribution of MT1 melatonin receptor promoter-driven RFP expression in the brains of BAC C3H/HeN transgenic mice. J Histochem Cytochem 62(1):70–84. 10.1369/0022155413507453 24051358 10.1369/0022155413507453 PMC3873804

[CR246] Adi N, Mash DC, Ali Y, Singer C, Shehadeh L, Papapetropoulos S (2010) Melatonin MT1 and MT2 receptor expression in Parkinsons disease. Med Sci Monit 16(2):61–67 20110911

[CR4] Ahmed J, Robertson NJ, More K (2021) Melatonin for neuroprotection in neonatal encephalopathy: a systematic review & meta-analysis of clinical trials. Eur J Paediatr Neurol 31:38–45 33601197 10.1016/j.ejpn.2021.02.003

[CR5] Akgun-Unal N, Ozyildirim S, Unal O, Gulbahce-Mutlu E, Mogulkoc R, Baltaci AK (2023) The effects of resveratrol and melatonin on biochemical and molecular parameters in diabetic old female rat hearts. Exp Gerontol 172:112043 36494013 10.1016/j.exger.2022.112043

[CR6] Albertini C, Salerno A (2021) From combinations to multitarget-directed ligands: a continuum in Alzheimer’s disease polypharmacology. Med Res Rev 41(5):2606–2633. 10.1002/med.21699 32557696 10.1002/med.21699

[CR7] Albrecht U (2012) Timing to perfection: the biology of central and peripheral circadian clocks. Neuron 74(2):246–260. 10.1016/j.neuron.2012.04.006 22542179 10.1016/j.neuron.2012.04.006

[CR8] Alghamdi BS, AboTaleb HA (2020) Melatonin improves memory defects in a mouse model of multiple sclerosis by up-regulating cAMP-response element-binding protein and synapse-associated proteins in the prefrontal cortex. J Integr Neurosci 19(2):229–237. 10.31083/j.jin.2020.02.32 32706187 10.31083/j.jin.2020.02.32

[CR9] Andersen LPH, Gögenur I, Rosenberg J, Reiter RJ (2016) The safety of melatonin in humans. Clin Drug Investig 36:169–175 26692007 10.1007/s40261-015-0368-5

[CR10] Anderson KN, Jamieson S, Graham AJ, Shneerson JM (2008) REM sleep behaviour disorder treated with melatonin in a patient with Alzheimer’s disease. Clin Neurol Neurosurg 110(5):492–495. 10.1016/j.clineuro.2008.01.004 18299172 10.1016/j.clineuro.2008.01.004

[CR11] Ando K, De Decker R, Vergara C, Yilmaz Z, Mansour S, Suain V, Sleegers K, de Fisenne MA, Houben S, Potier MC, Duyckaerts C, Watanabe T, Buée L, Leroy K, Brion JP (2020) Picalm reduction exacerbates tau pathology in a murine tauopathy model. Acta Neuropathol 139(4):773–789. 10.1007/s00401-020-02125-x 31925534 10.1007/s00401-020-02125-x

[CR12] Anghel L, Baroiu L, Popazu CR, Pătraș D, Fotea S, Nechifor A, Ciubara A, Nechita L, Mușat CL, Stefanopol IA (2022) Benefits and adverse events of melatonin use in the elderly. Exp Ther Med 23(3):219 35126722 10.3892/etm.2022.11142 PMC8796282

[CR13] Anwal L (2021) A comprehensive review on Alzheimer’s disease. World J Pharm Pharm Sci 10(7):1170

[CR14] Armstrong SM, Redman JR (1991) Melatonin: a chronobiotic with anti-aging properties? Med Hypotheses 34(4):300–309. 10.1016/0306-9877(91)90046-2 1865836 10.1016/0306-9877(91)90046-2

[CR15] Asai M, Yoshinobu Y, Kaneko S, Mori A, Nikaido T, Moriya T, Akiyama M, Shibata S (2001) Circadian profile of per gene mRNA expression in the suprachiasmatic nucleus, paraventricular nucleus, and pineal body of aged rats. J Neurosci Res 66(6):1133–1139. 10.1002/jnr.10010 11746446 10.1002/jnr.10010

[CR16] Asma A, Marc-André S (2022) Melatonin signaling pathways implicated in metabolic processes in human granulosa cells (KGN). Int J Mol Sci 23(6):2988 35328408 10.3390/ijms23062988 PMC8950389

[CR17] Atacak A, Baltaci SB, Akgun-Unal N, Mogulkoc R, Baltaci AK (2023) Melatonin protects retinal tissue damage in streptozotocin-induced aged rats. Arch Gerontol Geriatr 112:105035 37075585 10.1016/j.archger.2023.105035

[CR18] Ballard C, Gauthier S, Corbett A, Brayne C, Aarsland D, Jones E (2011) Alzheimer’s disease. Lancet 377(9770):1019–1031. 10.1016/s0140-6736(10)61349-9 21371747 10.1016/S0140-6736(10)61349-9

[CR19] Banerjee R, Purhonen J, Kallijärvi J (2022) The mitochondrial coenzyme Q junction and complex III: biochemistry and pathophysiology. FEBS J 289(22):6936–6958 34428349 10.1111/febs.16164

[CR20] Barnes DE, Yaffe K (2011) The projected effect of risk factor reduction on Alzheimer’s disease prevalence. Lancet Neurol 10(9):819–828. 10.1016/s1474-4422(11)70072-2 21775213 10.1016/S1474-4422(11)70072-2 PMC3647614

[CR21] Besag FM, Vasey MJ (2022) Adverse events in long-term studies of exogenous melatonin. Exp Opin Drug Saf 21(12):1469–1481 10.1080/14740338.2022.2160444 36562403

[CR22] Besancon E, Guo S, Lok J, Tymianski M, Lo EH (2008) Beyond NMDA and AMPA glutamate receptors: emerging mechanisms for ionic imbalance and cell death in stroke. Trends Pharmacol Sci 29(5):268–275. 10.1016/j.tips.2008.02.003 18384889 10.1016/j.tips.2008.02.003

[CR23] Bettonte S, Berton M, Marzolini C (2022) Magnitude of drug-drug interactions in special populations. Pharmaceutics. 10.3390/pharmaceutics14040789 35456623 10.3390/pharmaceutics14040789 PMC9027396

[CR24] Bhattacharya S, Patel KK, Dehari D, Agrawal AK, Singh S (2019) Melatonin and its ubiquitous anticancer effects. Mol Cell Biochem 462:133–155 31451998 10.1007/s11010-019-03617-5

[CR25] Bjedov I, Rallis C (2020) The target of rapamycin signalling pathway in ageing and lifespan regulation. Genes 11(9):1043 32899412 10.3390/genes11091043 PMC7565554

[CR26] Bjelakovic G, Nikolova D, Gluud LL, Simonetti RG, Gluud C (2007) Mortality in randomized trials of antioxidant supplements for primary and secondary prevention: systematic review and meta-analysis. JAMA 297(8):842–857. 10.1001/jama.297.8.842 17327526 10.1001/jama.297.8.842

[CR27] Blask DE, Sauer LA, Dauchy RT, Holowachuk EW, Ruhoff MS, Kopff HS (1999) Melatonin inhibition of cancer growth in vivo involves suppression of tumor fatty acid metabolism via melatonin receptor-mediated signal transduction events. Cancer Res 59(18):4693–4701 10493527

[CR28] Bliwise DL, Mercaldo ND, Avidan AY, Boeve BF, Greer SA, Kukull WA (2011) Sleep disturbance in dementia with Lewy bodies and Alzheimer’s disease: a multicenter analysis. Dement Geriatr Cogn Disord 31(3):239–246. 10.1159/000326238 21474933 10.1159/000326238 PMC3085031

[CR29] Bohnen NI, Kaufer DI, Ivanco LS, Lopresti B, Koeppe RA, Davis JG, Mathis CA, Moore RY, DeKosky ST (2003) Cortical cholinergic function is more severely affected in parkinsonian dementia than in Alzheimer disease: an in vivo positron emission tomographic study. Arch Neurol 60(12):1745–1748. 10.1001/archneur.60.12.1745 14676050 10.1001/archneur.60.12.1745

[CR30] Boland B, Yu WH, Corti O, Mollereau B, Henriques A, Bezard E, Pastores GM, Rubinsztein DC, Nixon RA, Duchen MR, Mallucci GR, Kroemer G, Levine B, Eskelinen EL, Mochel F, Spedding M, Louis C, Martin OR, Millan MJ (2018) Promoting the clearance of neurotoxic proteins in neurodegenerative disorders of ageing. Nat Rev Drug Discov 17(9):660–688. 10.1038/nrd.2018.109 30116051 10.1038/nrd.2018.109 PMC6456907

[CR31] Bondy SC, Campbell A (2020) Melatonin and regulation of immune function: impact on numerous diseases. Curr Aging Sci 13(2):92–101 32651969 10.2174/1874609813666200711153223

[CR32] Bonmati-Carrion MA, Arguelles-Prieto R, Martinez-Madrid MJ, Reiter R, Hardeland R, Rol MA, Madrid JA (2014) Protecting the melatonin rhythm through circadian healthy light exposure. Int J Mol Sci 15(12):23448–23500. 10.3390/ijms151223448 25526564 10.3390/ijms151223448 PMC4284776

[CR33] Bonomini F, Borsani E, Favero G, Rodella LF, Rezzani R (2018) Dietary melatonin supplementation could be a promising preventing/therapeutic approach for a variety of liver diseases. Nutrients 10(9):1135 30134592 10.3390/nu10091135 PMC6164189

[CR34] Boyd CA (2001) Amine uptake and peptide hormone secretion: APUD cells in a new landscape. J Physiol 531(Pt 3):581. 10.1111/j.1469-7793.2001.0581h.x 11251039 10.1111/j.1469-7793.2001.0581h.x PMC2278499

[CR35] Bruns DR, Drake JC, Biela LM, Peelor FF III, Miller BF (2015) Hamilton KL (2015) Nrf2 signaling and the slowed aging phenotype: evidence from long-lived models. Oxid Med Cell Longev 1:732596 10.1155/2015/732596 PMC4637130 26583062

[CR36] Brusco LI, Márquez M, Cardinali DP (1998) Monozygotic twins with Alzheimer’s disease treated with melatonin: case report. J Pineal Res 25(4):260–263. 10.1111/j.1600-079x.1998.tb00396.x 9885996 10.1111/j.1600-079x.1998.tb00396.x

[CR37] Burgess HJ, Revell VL, Molina TA, Eastman CI (2010) Human phase response curves to three days of daily melatonin: 0.5 mg versus 3.0 mg. J Clin Endocrinol Metab 95(7):3325–3331. 10.1210/jc.2009-2590 20410229 10.1210/jc.2009-2590 PMC2928909

[CR38] Camell CD, Yousefzadeh MJ, Zhu Y, Prata LGL, Huggins MA, Pierson M, Zhang L, O’Kelly RD, Pirtskhalava T, Xun P (2021) Senolytics reduce coronavirus-related mortality in old mice. Science 373(6552):eabe4832 34103349 10.1126/science.abe4832 PMC8607935

[CR39] Campbell FC, Xu H, El-Tanani M, Crowe P, Bingham V (2010) The yin and yang of vitamin D receptor (VDR) signaling in neoplastic progression: operational networks and tissue-specific growth control. Biochem Pharmacol 79(1):1–9. 10.1016/j.bcp.2009.09.005 19737544 10.1016/j.bcp.2009.09.005 PMC2824849

[CR40] Cantarero G, Tang B, O’Malley R, Salas R, Celnik P (2013) Motor learning interference is proportional to occlusion of LTP-like plasticity. J Neurosci 33(11):4634–4641 23486938 10.1523/JNEUROSCI.4706-12.2013 PMC3727291

[CR41] Cardinali DP (2019) Melatonin: clinical perspectives in neurodegeneration. Front Endocrinol (Lausanne) 10:480. 10.3389/fendo.2019.00480 31379746 10.3389/fendo.2019.00480 PMC6646522

[CR42] Carrillo-Vico A, Lardone PJ, Alvarez-Sánchez N, Rodríguez-Rodríguez A, Guerrero JM (2013) Melatonin: buffering the immune system. Int J Mol Sci 14(4):8638–8683. 10.3390/ijms14048638 23609496 10.3390/ijms14048638 PMC3645767

[CR43] Casimiro MC, Crosariol M, Loro E, Li Z, Pestell RG (2012) Cyclins and cell cycle control in cancer and disease. Genes Cancer 3(11–12):649–657. 10.1177/1947601913479022 23634253 10.1177/1947601913479022 PMC3636749

[CR44] Ceravolo R, Volterrani D, Frosini D, Bernardini S, Rossi C, Logi C, Manca G, Kiferle L, Mariani G, Murri L, Bonuccelli U (2006) Brain perfusion effects of cholinesterase inhibitors in Parkinson’s disease with dementia. J Neural Transm (Vienna) 113(11):1787–1790. 10.1007/s00702-006-0478-6 16758132 10.1007/s00702-006-0478-6

[CR45] Cerella C, Grandjenette C, Dicato M, Diederich M (2016) Roles of apoptosis and cellular senescence in cancer and aging. Curr Drug Targets 17(4):405–415 25642721 10.2174/1389450116666150202155915

[CR46] Cermakian N, Boivin DB (2009) The regulation of central and peripheral circadian clocks in humans. Obes Rev 10(Suppl 2):25–36. 10.1111/j.1467-789X.2009.00660.x 19849799 10.1111/j.1467-789X.2009.00660.x

[CR47] Chang J, Jiang L, Wang Y, Yao B, Yang S, Zhang B, Zhang MZ (2015) 12/15 Lipoxygenase regulation of colorectal tumorigenesis is determined by the relative tumor levels of its metabolite 12-HETE and 13-HODE in animal models. Oncotarget 6(5):2879–2888. 10.18632/oncotarget.2994 25576922 10.18632/oncotarget.2994 PMC4413624

[CR48] Chang JT, Kumsta C, Hellman AB, Adams LM, Hansen M (2017) Spatiotemporal regulation of autophagy during Caenorhabditis elegans aging. Elife. 10.7554/eLife.18459 28675140 10.7554/eLife.18459 PMC5496740

[CR49] Chen D, Zhang T, Lee TH (2020a) Cellular mechanisms of melatonin: insight from neurodegenerative diseases. Biomolecules 10(8):1158 32784556 10.3390/biom10081158 PMC7464852

[CR50] Chen M, Cecon E, Karamitri A, Gao W, Gerbier R, Ahmad R, Jockers R (2020b) Melatonin MT1 and MT2 receptor ERK signaling is differentially dependent on Gi/o and Gq/11 proteins. J Pineal Res 68(4):e12641 32080899 10.1111/jpi.12641

[CR51] Chen X, Gong W, Shao X, Shi T, Zhang L, Dong J, Shi Y, Shen S, Qin J, Jiang Q, Guo B (2022) METTL3-mediated m(6)A modification of ATG7 regulates autophagy-GATA4 axis to promote cellular senescence and osteoarthritis progression. Ann Rheum Dis 81(1):87–99. 10.1136/annrheumdis-2021-221091 34706873 10.1136/annrheumdis-2021-221091

[CR52] Chern CM, Liao JF, Wang YH, Shen YC (2012) Melatonin ameliorates neural function by promoting endogenous neurogenesis through the MT2 melatonin receptor in ischemic-stroke mice. Free Radic Biol Med 52(9):1634–1647. 10.1016/j.freeradbiomed.2012.01.030 22330064 10.1016/j.freeradbiomed.2012.01.030

[CR53] Choksi KB, Nuss JE, Boylston WH, Rabek JP, Papaconstantinou J (2007) Age-related increases in oxidatively damaged proteins of mouse kidney mitochondrial electron transport chain complexes. Free Radical Biol Med 43(10):1423–1438 17936188 10.1016/j.freeradbiomed.2007.07.027 PMC2080815

[CR54] Chu CT (2019) Mechanisms of selective autophagy and mitophagy: Implications for neurodegenerative diseases. Neurobiol Dis 122:23–34. 10.1016/j.nbd.2018.07.015 30030024 10.1016/j.nbd.2018.07.015 PMC6396690

[CR55] Cong L, Liu X, Bai Y, Qin Q, Zhao L, Shi Y, Bai Y, Guo Z (2023) Melatonin alleviates pyroptosis by regulating the SIRT3/FOXO3α/ROS axis and interacting with apoptosis in Atherosclerosis progression. Biol Res 56(1):62. 10.1186/s40659-023-00479-6 38041171 10.1186/s40659-023-00479-6 PMC10693060

[CR56] Cook DN, Kang HS, Jetten AM (2015) Retinoic acid-related orphan receptors (RORs): regulatory functions in immunity, development, circadian rhythm, and metabolism. Nucl Receptor Res. 10.11131/2015/101185 26878025 10.11131/2015/101185 PMC4750502

[CR57] Corrales A, Martínez P, García S, Vidal V, García E, Flórez J, Sanchez-Barceló EJ, Martínez-Cué C, Rueda N (2013) Long-term oral administration of melatonin improves spatial learning and memory and protects against cholinergic degeneration in middle-aged Ts65Dn mice, a model of Down syndrome. J Pineal Res 54(3):346–358. 10.1111/jpi.12037 23350971 10.1111/jpi.12037

[CR58] Cortes-Canteli M, Iadecola C (2020) Alzheimer’s disease and vascular aging: JACC focus seminar. J Am Coll Cardiol 75(8):942–951. 10.1016/j.jacc.2019.10.062 32130930 10.1016/j.jacc.2019.10.062 PMC8046164

[CR59] Cruz-Sanabria F, Carmassi C, Bruno S, Bazzani A, Carli M, Scarselli M, Faraguna U (2023) Melatonin as a chronobiotic with sleep-promoting properties. Curr Neuropharmacol 21(4):951–987 35176989 10.2174/1570159X20666220217152617 PMC10227911

[CR60] Cutando A, Aneiros-Fernández J, López-Valverde A, Arias-Santiago S, Aneiros-Cachaza J, Reiter RJ (2011) A new perspective in Oral health: potential importance and actions of melatonin receptors MT1, MT2, MT3, and RZR/ROR in the oral cavity. Arch Oral Biol 56(10):944–950. 10.1016/j.archoralbio.2011.03.004 21459362 10.1016/j.archoralbio.2011.03.004

[CR61] Cutler NR, Polinsky RJ, Sramek JJ, Enz A, Jhee SS, Mancione L, Hourani J, Zolnouni P (1998) Dose-dependent CSF acetylcholinesterase inhibition by SDZ ENA 713 in Alzheimer’s disease. Acta Neurol Scand 97(4):244–250. 10.1111/j.1600-0404.1998.tb00645.x 9576639 10.1111/j.1600-0404.1998.tb00645.x

[CR62] Darreh-Shori T, Almkvist O, Guan ZZ, Garlind A, Strandberg B, Svensson AL, Soreq H, Hellström-Lindahl E, Nordberg A (2002) Sustained cholinesterase inhibition in AD patients receiving rivastigmine for 12 months. Neurology 59(4):563–572. 10.1212/wnl.59.4.563 12196650 10.1212/wnl.59.4.563

[CR63] Davidson AJ, Yamazaki S, Arble DM, Menaker M, Block GD (2008) Resetting of central and peripheral circadian oscillators in aged rats. Neurobiol Aging 29(3):471–477. 10.1016/j.neurobiolaging.2006.10.018 17129640 10.1016/j.neurobiolaging.2006.10.018 PMC1635489

[CR64] de Bodinat C, Guardiola-Lemaitre B, Mocaër E, Renard P, Muñoz C, Millan MJ (2010) Agomelatine, the first melatonergic antidepressant: discovery, characterization and development. Nat Rev Drug Discov 9(8):628–642. 10.1038/nrd3140 20577266 10.1038/nrd3140

[CR65] De Pace R, Skirzewski M, Damme M, Mattera R, Mercurio J, Foster AM, Cuitino L, Jarnik M, Hoffmann V, Morris HD, Han TU, Mancini GMS, Buonanno A, Bonifacino JS (2018) Altered distribution of ATG9A and accumulation of axonal aggregates in neurons from a mouse model of AP-4 deficiency syndrome. PLoS Genet 14(4):e1007363. 10.1371/journal.pgen.1007363 29698489 10.1371/journal.pgen.1007363 PMC5940238

[CR66] Ding J, Lohman K, Molina A, Delbono O, Bertoni A, Shea S, Post W, Guo X, Barr RG, Manichaikul AW, Pankow JS, Rotter JI, Hoeschele I, Kritchevsky SB, Liu Y (2023) The association between aging-related monocyte transcriptional networks and comorbidity burden: the Multi-Ethnic Study of Atherosclerosis (MESA). Geroscience 45(1):197–207. 10.1007/s11357-022-00608-1 35737188 10.1007/s11357-022-00608-1 PMC9886705

[CR67] Dubocovich ML, Delagrange P, Krause DN, Sugden D, Cardinali DP, Olcese J (2010) International union of basic and clinical pharmacology. LXXV. Nomenclature, classification, and pharmacology of G protein-coupled melatonin receptors. Pharmacol Rev 62(3):343–380. 10.1124/pr.110.002832 20605968 10.1124/pr.110.002832 PMC2964901

[CR68] Dubowy C, Sehgal A (2017) Circadian rhythms and sleep in Drosophila melanogaster. Genetics 205(4):1373–1397 28360128 10.1534/genetics.115.185157 PMC5378101

[CR69] Dubrovsky YV, Samsa WE, Kondratov RV (2010) Deficiency of circadian protein CLOCK reduces lifespan and increases age-related cataract development in mice. Aging (Albany NY) 2(12):936–944. 10.18632/aging.100241 21149897 10.18632/aging.100241 PMC3034182

[CR70] Ebadi M, Govitrapong P, Sharma S, Muralikrishnan D, Shavali S, Pellett L, Schafer R, Albano C, Eken J (2001) Ubiquinone (coenzyme q10) and mitochondria in oxidative stress of Parkinson’s disease. Neurosignals 10(3–4):224–253 10.1159/000046889 11351130

[CR71] Emet M, Ozcan H, Ozel L, Yayla M, Halici Z, Hacimuftuoglu A (2016) A review of melatonin, its receptors and drugs. Eurasian J Med 48(2):135–141. 10.5152/eurasianjmed.2015.0267 27551178 10.5152/eurasianjmed.2015.0267 PMC4970552

[CR72] Eslamizade MJ, Madjd Z, Rasoolijazi H, Saffarzadeh F, Pirhajati V, Aligholi H, Janahmadi M, Mehdizadeh M (2016) Impaired memory and evidence of histopathology in CA1 pyramidal neurons through injection of Aβ1-42 peptides into the frontal cortices of rat. Basic Clin Neurosci 7(1):31–42 27303597 PMC4892328

[CR73] Feng Y, Jiang X, Liu W, Lu H (2023) The location, physiology, pathology of hippocampus Melatonin MT(2) receptor and MT(2)-selective modulators. Eur J Med Chem 262:115888. 10.1016/j.ejmech.2023.115888 37866336 10.1016/j.ejmech.2023.115888

[CR74] Fernandes SA, Demetriades C (2021) The multifaceted role of nutrient sensing and mTORC1 signaling in physiology and aging. Front Aging 2:707372 35822019 10.3389/fragi.2021.707372 PMC9261424

[CR75] Fodale V, Quattrone D, Trecroci C, Caminiti V, Santamaria LB (2006) Alzheimer’s disease and anaesthesia: implications for the central cholinergic system. Br J Anaesth 97(4):445–452. 10.1093/bja/ael233 16950812 10.1093/bja/ael233

[CR76] Fogelson N, Kogan E, Korczyn AD, Giladi N, Shabtai H, Neufeld MY (2003) Effects of rivastigmine on the quantitative EEG in demented Parkinsonian patients. Acta Neurol Scand 107(4):252–255. 10.1034/j.1600-0404.2003.00081.x 12675697 10.1034/j.1600-0404.2003.00081.x

[CR77] Frake RA, Ricketts T, Menzies FM, Rubinsztein DC (2015) Autophagy and neurodegeneration. J Clin Invest 125(1):65–74. 10.1172/jci73944 25654552 10.1172/JCI73944 PMC4382230

[CR78] Galano A, Tan DX, Reiter RJ (2011) Melatonin as a natural ally against oxidative stress: a physicochemical examination. J Pineal Res 51(1):1–16 21752095 10.1111/j.1600-079X.2011.00916.x

[CR79] Galano A, Tan DX, Reiter RJ (2013) On the free radical scavenging activities of melatonin’s metabolites, AFMK and AMK. J Pineal Res 54(3):245–257. 10.1111/jpi.12010 22998574 10.1111/jpi.12010

[CR249] Gans P, Sabatini A, Vacca A (1996) Investigation of equilibria in solution. Determination of equilibrium constants with the hyperquad suite of programs. Talanta 43(10):1739–1753. 10.1016/0039-9140(96)01958-3 18966661 10.1016/0039-9140(96)01958-3

[CR80] García T, Ribes D, Colomina MT, Cabré M, Domingo JL, Gómez M (2009) Evaluation of the protective role of melatonin on the behavioral effects of aluminum in a mouse model of Alzheimer’s disease. Toxicology 265(1):49–55. 10.1016/j.tox.2009.09.009 19770021 10.1016/j.tox.2009.09.009

[CR81] Genova ML, Lenaz G (2015) The interplay between respiratory supercomplexes and ROS in aging. Antioxid Redox Signal 23(3):208–238 25711676 10.1089/ars.2014.6214

[CR82] Givler D, Givler A, Luther PM, Wenger DM, Ahmadzadeh S, Shekoohi S, Edinoff AN, Dorius BK, Jean Baptiste C, Cornett EM (2023) Chronic administration of melatonin: physiological and clinical considerations. Neurol Int 15(1):518–533 36976674 10.3390/neurolint15010031 PMC10053496

[CR83] Gómez LA, Hagen TM (2012) Age-related decline in mitochondrial bioenergetics: does supercomplex destabilization determine lower oxidative capacity and higher superoxide production? Seminars in cell & developmental biology. Elsevier, Amsterdam, pp 758–767 10.1016/j.semcdb.2012.04.002 PMC4096948 22521482

[CR84] Greig NH, Utsuki T, Ingram DK, Wang Y, Pepeu G, Scali C, Yu QS, Mamczarz J, Holloway HW, Giordano T, Chen D, Furukawa K, Sambamurti K, Brossi A, Lahiri DK (2005) Selective butyrylcholinesterase inhibition elevates brain acetylcholine, augments learning and lowers Alzheimer beta-amyloid peptide in rodent. Proc Natl Acad Sci USA 102(47):17213–17218. 10.1073/pnas.0508575102 16275899 10.1073/pnas.0508575102 PMC1288010

[CR85] Grunewald M, Kumar S, Sharife H, Volinsky E, Gileles-Hillel A, Licht T, Permyakova A, Hinden L, Azar S, Friedmann Y, Kupetz P, Tzuberi R, Anisimov A, Alitalo K, Horwitz M, Leebhoff S, Khoma OZ, Hlushchuk R, Djonov V, Keshet E (2021) Counteracting age-related VEGF signaling insufficiency promotes healthy aging and extends life span. Science. 10.1126/science.abc8479 34326210 10.1126/science.abc8479

[CR86] Guerrero JM, Reiter RJ (2002) Melatonin-immune system relationships. Curr Top Med Chem 2(2):167–179. 10.2174/1568026023394335 11899099 10.2174/1568026023394335

[CR87] Guiselin T, Lecoutey C, Rochais C, Dallemagne P (2023) Conceptual framework of the design of pleiotropic drugs against Alzheimer’s disease. Pharmaceutics. 10.3390/pharmaceutics15102382 37896142 10.3390/pharmaceutics15102382 PMC10610275

[CR88] Hadi A, Ghaedi E, Moradi S, Pourmasoumi M, Ghavami A, Kafeshani M (2019) Effects of melatonin supplementation on blood pressure: a systematic review and meta-analysis of randomized controlled trials. Horm Metab Res 51(03):157–164 30861561 10.1055/a-0841-6638

[CR89] Hardeland R (2012) Neurobiology, pathophysiology, and treatment of melatonin deficiency and dysfunction. ScientificWorldJournal 2012:640389. 10.1100/2012/640389 22629173 10.1100/2012/640389 PMC3354573

[CR90] Hardeland R (2016) Melatonin and synthetic melatoninergic agonists in psychiatric and age-associated disorders: successful and unsuccessful approaches. Curr Pharm des 22(8):1086–1101 25248806 10.2174/1381612822666151214125543

[CR91] Hardeland R (2017) Melatonin and the electron transport chain. Cell Mol Life Sci 74:3883–3896 28785805 10.1007/s00018-017-2615-9 PMC11107625

[CR92] Hardeland R (2021) Melatonin, its metabolites and their interference with reactive nitrogen compounds. Molecules 26(13):4105 34279445 10.3390/molecules26134105 PMC8271479

[CR93] Hardeland R, Pandi-Perumal SR (2005) Melatonin, a potent agent in antioxidative defense: actions as a natural food constituent, gastrointestinal factor, drug and prodrug. Nutr Metab (Lond) 2:22. 10.1186/1743-7075-2-22 16153306 10.1186/1743-7075-2-22 PMC1262766

[CR94] Harper DG, Stopa EG, McKee AC, Satlin A, Fish D, Volicer L (2004) Dementia severity and Lewy bodies affect circadian rhythms in Alzheimer disease. Neurobiol Aging 25(6):771–781. 10.1016/j.neurobiolaging.2003.04.009 15165702 10.1016/j.neurobiolaging.2003.04.009

[CR95] Hartley S, Dauvilliers Y, Quera-Salva MA (2018) Circadian rhythm disturbances in the blind. Curr Neurol Neurosci Rep 18:1–8 30083814 10.1007/s11910-018-0876-9

[CR96] Hasan MK, Liu CX, Pan YT, Ahammed GJ, Qi ZY, Zhou J (2018) Melatonin alleviates low-sulfur stress by promoting sulfur homeostasis in tomato plants. Sci Rep 8(1):10182. 10.1038/s41598-018-28561-0 29976982 10.1038/s41598-018-28561-0 PMC6033901

[CR97] He L, He T, Farrar S, Ji L, Liu T, Ma X (2017) Antioxidants maintain cellular redox homeostasis by elimination of reactive oxygen species. Cell Physiol Biochem 44(2):532–553. 10.1159/000485089 29145191 10.1159/000485089

[CR98] He F, Ru X, Wen T (2020) NRF2, a transcription factor for stress response and beyond. Int J Mol Sci 21(13):4777 32640524 10.3390/ijms21134777 PMC7369905

[CR99] Hernández Cordero AI, Yang CX, Yang J, Horvath S, Shaipanich T, MacIsaac J, Lin DTS, Kobor MS, Guillemi S, Harris M, Lam W, Lam S, Montaner J, Man SFP, Sin DD, Leung JM (2022) Airway aging and methylation disruptions in HIV-associated chronic obstructive pulmonary disease. Am J Respir Crit Care Med 206(2):150–160. 10.1164/rccm.202106-1440OC 35426765 10.1164/rccm.202106-1440OC PMC9887412

[CR100] Hiremathad A, Keri RS, Esteves AR, Cardoso SM, Chaves S, Santos MA (2018) Novel tacrine-hydroxyphenylbenzimidazole hybrids as potential multitarget drug candidates for Alzheimer’s disease. Eur J Med Chem 148:255–267. 10.1016/j.ejmech.2018.02.023 29466775 10.1016/j.ejmech.2018.02.023

[CR101] Hoehn MM, Yahr MD (1967) Parkinsonism: onset, progression and mortality. Neurology 17(5):427–442. 10.1212/wnl.17.5.427 6067254 10.1212/wnl.17.5.427

[CR102] Hussain F, Kayani HUR (2020) Aging-Oxidative stress, antioxidants and computational modeling. Heliyon 6(5):e04107 32509998 10.1016/j.heliyon.2020.e04107 PMC7264715

[CR103] Jacob S, Poeggeler B, Weishaupt JH, Sirén AL, Hardeland R, Bähr M, Ehrenreich H (2002) Melatonin as a candidate compound for neuroprotection in amyotrophic lateral sclerosis (ALS): high tolerability of daily oral melatonin administration in ALS patients. J Pineal Res 33(3):186–187. 10.1034/j.1600-079x.2002.02943.x 12220335 10.1034/j.1600-079x.2002.02943.x

[CR104] Jurcau A (2021) Insights into the pathogenesis of neurodegenerative diseases: focus on mitochondrial dysfunction and oxidative stress. Int J Mol Sci 22(21):11847 34769277 10.3390/ijms222111847 PMC8584731

[CR105] Jürgenson M, Zharkovskaja T, Noortoots A, Morozova M, Beniashvili A, Zapolski M, Zharkovsky A (2019) Effects of the drug combination memantine and melatonin on impaired memory and brain neuronal deficits in an amyloid-predominant mouse model of Alzheimer’s disease. J Pharm Pharmacol 71(11):1695–1705. 10.1111/jphp.13165 31531878 10.1111/jphp.13165

[CR106] Kakhaki RD, Ostadmohammadi V, Kouchaki E, Aghadavod E, Bahmani F, Tamtaji OR, Reiter RJ, Mansournia MA, Asemi Z (2020) Melatonin supplementation and the effects on clinical and metabolic status in Parkinson’s disease: a randomized, double-blind, placebo-controlled trial. Clin Neurol Neurosurg 195:105878 32417629 10.1016/j.clineuro.2020.105878

[CR107] Karasek M (2004) Melatonin, human aging, and age-related diseases. Exp Gerontol 39(11–12):1723–1729. 10.1016/j.exger.2004.04.012 15582288 10.1016/j.exger.2004.04.012

[CR108] Karunanithi D, Radhakrishna A, Sivaraman KP, Biju VM (2014) Quantitative determination of melatonin in milk by LC-MS/MS. J Food Sci Technol 51(4):805–812. 10.1007/s13197-013-1221-6 24741180 10.1007/s13197-013-1221-6 PMC3981994

[CR109] Kaufman P, Poliakoff H (1950) Studies on the aging heart; the pattern of rheumatic heart disease in old age (a clinical pathological study). Ann Intern Med 32(5):889–904. 10.7326/0003-4819-32-5-889 15413908 10.7326/0003-4819-32-5-889

[CR110] Kennaway DJ (2022) What do we really know about the safety and efficacy of melatonin for sleep disorders? Curr Med Res Opin 38(2):211–227 34714707 10.1080/03007995.2021.2000714

[CR111] Khachiyants N, Trinkle D, Son SJ, Kim KY (2011) Sundown syndrome in persons with dementia: an update. Psychiatry Investig 8(4):275–287. 10.4306/pi.2011.8.4.275 22216036 10.4306/pi.2011.8.4.275 PMC3246134

[CR112] Klass MR (1983) A method for the isolation of longevity mutants in the nematode Caenorhabditis elegans and initial results. Mech Ageing Dev 22(3–4):279–286. 10.1016/0047-6374(83)90082-9 6632998 10.1016/0047-6374(83)90082-9

[CR113] Kochergin IA, Shpilyukova YA, Lysogorskaia EV, Abramycheva NY, Zakharova MN, Illarioshkin SN (2019) Effect of Mutations in SOD1 and C9orf72 Genes on Autophagy in Lymphomonocytes in Myotrophic Lateral Sclerosis. Bull Exp Biol Med 167(5):667–670. 10.1007/s10517-019-04595-w 31625064 10.1007/s10517-019-04595-w

[CR114] Kolker DE, Fukuyama H, Huang DS, Takahashi JS, Horton TH, Turek FW (2003) Aging alters circadian and light-induced expression of clock genes in golden hamsters. J Biol Rhythms 18(2):159–169. 10.1177/0748730403251802 12693870 10.1177/0748730403251802

[CR115] Kondratova AA, Kondratov RV (2012) The circadian clock and pathology of the ageing brain. Nat Rev Neurosci 13(5):325–335. 10.1038/nrn3208 22395806 10.1038/nrn3208 PMC3718301

[CR116] Kuciel-Lewandowska J, Kasperczak M, Bogut B, Heider R, Laber WT, Laber W, Paprocka-Borowicz M (2020) The Impact of Health Resort Treatment on the Nonenzymatic Endogenous Antioxidant System. Oxid Med Cell Longev 2020:8423105. 10.1155/2020/8423105 32089783 10.1155/2020/8423105 PMC7016389

[CR117] Labban S, Alghamdi BS, Alshehri FS, Kurdi M (2021a) Effects of melatonin and resveratrol on recognition memory and passive avoidance performance in a mouse model of Alzheimer’s disease. Behav Brain Res 402:113100. 10.1016/j.bbr.2020.113100 33417994 10.1016/j.bbr.2020.113100

[CR118] Labban S, Alshehri FS, Kurdi M, Alatawi Y, Alghamdi BS (2021) Melatonin improves short-term spatial memory in a mouse model of Alzheimer’s disease. Degenerative neurological and neuromuscular disease:15–27 10.2147/DNND.S291172 PMC8110255 33986623

[CR119] Lahiri DK, Chen D, Ge YW, Bondy SC, Sharman EH (2004) Dietary supplementation with melatonin reduces levels of amyloid beta-peptides in the murine cerebral cortex. J Pineal Res 36(4):224–231. 10.1111/j.1600-079X.2004.00121.x 15066046 10.1111/j.1600-079X.2004.00121.x

[CR120] Lammers M, Ahmed AIA (2013) Melatonin for sundown syndrome and delirium in dementia: is it effective? J Am Geriatr Soc 61(6):1045–1046. 10.1111/jgs.12296 23772740 10.1111/jgs.12296

[CR121] Lamptey RNL, Chaulagain B, Trivedi R, Gothwal A, Layek B, Singh J (2022) A review of the common neurodegenerative disorders: current therapeutic approaches and the potential role of nanotherapeutics. Int J Mol Sci. 10.3390/ijms23031851 35163773 10.3390/ijms23031851 PMC8837071

[CR122] Lapin V, Ebels I (1976) Effects of some low molecular weight sheep pineal fractions and melatonin on different tumors in rats and mice. Oncology 33(3):110–113. 10.1159/000225117 1012644 10.1159/000225117

[CR123] Laudon M, Frydman-Marom A (2014) Therapeutic effects of melatonin receptor agonists on sleep and comorbid disorders. Int J Mol Sci 15(9):15924–15950. 10.3390/ijms150915924 25207602 10.3390/ijms150915924 PMC4200764

[CR124] Laurindo LF, Simili OAG, Araújo AC, Guiguer EL, Direito R, Valenti VE, de Oliveira V, de Oliveira JS, Yanaguizawa Junior JL, Dias JA (2025) Melatonin from plants: going beyond traditional central nervous system targeting—a comprehensive review of its unusual health benefits. Biology 14(2):143 40001911 10.3390/biology14020143 PMC11851571

[CR125] Leon-Blanco MM, Guerrero JM, Reiter RJ, Calvo JR, Pozo D (2003) Melatonin inhibits telomerase activity in the MCF-7 tumor cell line both in vivo and in vitro. J Pineal Res 35(3):204–211. 10.1034/j.1600-079x.2003.00077.x 12932205 10.1034/j.1600-079x.2003.00077.x

[CR126] Libowitz MR, Nurmi EL (2021) The burden of antipsychotic-induced weight gain and metabolic syndrome in children. Front Psych 12:623681 10.3389/fpsyt.2021.623681 PMC7994286 33776816

[CR127] Lie PPY, Nixon RA (2019) Lysosome trafficking and signaling in health and neurodegenerative diseases. Neurobiol Dis 122:94–105. 10.1016/j.nbd.2018.05.015 29859318 10.1016/j.nbd.2018.05.015 PMC6381838

[CR128] Lin L, Huang QX, Yang SS, Chu J, Wang JZ, Tian Q (2013) Melatonin in Alzheimer’s disease. Int J Mol Sci 14(7):14575–14593. 10.3390/ijms140714575 23857055 10.3390/ijms140714575 PMC3742260

[CR129] Lisman J, Cooper K, Sehgal M, Silva AJ (2018) Memory formation depends on both synapse-specific modifications of synaptic strength and cell-specific increases in excitability. Nat Neurosci 21(3):309–314 29434376 10.1038/s41593-018-0076-6 PMC5915620

[CR130] Liu J, Somera-Molina KC, Hudson RL, Dubocovich ML (2013) Melatonin potentiates running wheel-induced neurogenesis in the dentate gyrus of adult C3H/HeN mice hippocampus. J Pineal Res 54(2):222–231. 10.1111/jpi.12023 23190173 10.1111/jpi.12023 PMC3568494

[CR131] Liu J, Clough SJ, Hutchinson AJ, Adamah-Biassi EB, Popovska-Gorevski M, Dubocovich ML (2016) MT1 and MT2 melatonin receptors: a therapeutic perspective. Annu Rev Pharmacol Toxicol 56:361–383. 10.1146/annurev-pharmtox-010814-124742 26514204 10.1146/annurev-pharmtox-010814-124742 PMC5091650

[CR132] Liu W, Wang Y, Youdim MBH (2022) A novel neuroprotective cholinesterase-monoamine oxidase inhibitor for treatment of dementia and depression in Parkinson’s disease. Age Neurodegen Dis 2(1):1

[CR133] Lopes J, Arnosti D, Trosko JE, Tai MH, Zuccari D (2016) Melatonin decreases estrogen receptor binding to estrogen response elements sites on the OCT4 gene in human breast cancer stem cells. Genes Cancer 7(5–6):209–217. 10.18632/genesandcancer.107 27551335 10.18632/genesandcancer.107 PMC4979593

[CR134] Low TL, Choo FN, Tan SM (2020) The efficacy of melatonin and melatonin agonists in insomnia–an umbrella review. J Psychiatr Res 121:10–23 31715492 10.1016/j.jpsychires.2019.10.022

[CR135] Lv D, Cui PL, Yao SW, Xu YQ, Yang ZX (2012) Melatonin inhibits the expression of vascular endothelial growth factor in pancreatic cancer cells. Chin J Cancer Res 24(4):310–316. 10.3978/j.issn.1000-9604.2012.09.03 23358453 10.3978/j.issn.1000-9604.2012.09.03 PMC3551319

[CR136] Mannino G, Pernici C, Serio G, Gentile C, Bertea CM (2021) Melatonin and phytomelatonin: chemistry, biosynthesis, metabolism, distribution and bioactivity in plants and animals-an overview. Int J Mol Sci 22(18):9996. 10.3390/ijms22189996 34576159 10.3390/ijms22189996 PMC8469784

[CR137] Martín Giménez VM, de Las HN, Lahera V, Tresguerres JAF, Reiter RJ, Manucha W (2022) Melatonin as an anti-aging therapy for age-related cardiovascular and neurodegenerative diseases. Front Aging Neurosci 14:888292. 10.3389/fnagi.2022.888292 35721030 10.3389/fnagi.2022.888292 PMC9204094

[CR138] Martínez-Campa C, Alonso-González C, Mediavilla MD, Cos S, González A, Ramos S, Sánchez-Barceló EJ (2006) Melatonin inhibits both ER alpha activation and breast cancer cell proliferation induced by a metalloestrogen, cadmium. J Pineal Res 40(4):291–296. 10.1111/j.1600-079X.2006.00315.x 16635015 10.1111/j.1600-079X.2006.00315.x

[CR139] Martínez-Campa C, González A, Mediavilla MD, Alonso-González C, Alvarez-García V, Sánchez-Barceló EJ, Cos S (2009) Melatonin inhibits aromatase promoter expression by regulating cyclooxygenases expression and activity in breast cancer cells. Br J Cancer 101(9):1613–1619. 10.1038/sj.bjc.6605336 19773750 10.1038/sj.bjc.6605336 PMC2778514

[CR140] Matsubara E, Bryant-Thomas T, Pacheco Quinto J, Henry TL, Poeggeler B, Herbert D, Cruz-Sanchez F, Chyan YJ, Smith MA, Perry G, Shoji M, Abe K, Leone A, Grundke-Ikbal I, Wilson GL, Ghiso J, Williams C, Refolo LM, Pappolla MA, Chain DG, Neria E (2003) Melatonin increases survival and inhibits oxidative and amyloid pathology in a transgenic model of Alzheimer’s disease. J Neurochem 85(5):1101–1108. 10.1046/j.1471-4159.2003.01654.x 12753069 10.1046/j.1471-4159.2003.01654.x

[CR141] Mattis J, Sehgal A (2016) Circadian rhythms, sleep, and disorders of aging. Trends Endocrinol Metab 27(4):192–203 26947521 10.1016/j.tem.2016.02.003 PMC4808513

[CR142] McCay CM, Maynard LA, Sperling G, Barnes LL (1975) The journal of nutrition. Retarded growth, life span, ultimate body size and age changes in the albino rat after feeding diets restricted in calories. Nutr Rev 33(8):241–243. 10.1111/j.1753-4887.1975.tb05227.x 1095975 10.1111/j.1753-4887.1975.tb05227.x

[CR143] Mealer RG, Murray AJ, Shahani N, Subramaniam S, Snyder SH (2014) Rhes, a striatal-selective protein implicated in Huntington disease, binds beclin-1 and activates autophagy. J Biol Chem 289(6):3547–3554. 10.1074/jbc.M113.536912 24324270 10.1074/jbc.M113.536912 PMC3916556

[CR144] Mediavilla MD, Sanchez-Barcelo EJ, Tan DX, Manchester L, Reiter RJ (2010) Basic mechanisms involved in the anti-cancer effects of melatonin. Curr Med Chem 17(36):4462–4481. 10.2174/092986710794183015 21062257 10.2174/092986710794183015

[CR145] Mesulam MM, Guillozet A, Shaw P, Levey A, Duysen EG, Lockridge O (2002) Acetylcholinesterase knockouts establish central cholinergic pathways and can use butyrylcholinesterase to hydrolyze acetylcholine. Neuroscience 110(4):627–639. 10.1016/s0306-4522(01)00613-3 11934471 10.1016/s0306-4522(01)00613-3

[CR146] Miller CJ, Gounder SS, Kannan S, Goutam K, Muthusamy VR, Firpo MA, Symons JD, Paine R III, Hoidal JR (1822) Rajasekaran NS (2012) Disruption of Nrf2/ARE signaling impairs antioxidant mechanisms and promotes cell degradation pathways in aged skeletal muscle. Biochim Et Biophys Acta Mol Basis Dis 6:1038–1050 10.1016/j.bbadis.2012.02.007 22366763

[CR147] Miwa S, Kashyap S, Chini E, von Zglinicki T (2022) Mitochondrial dysfunction in cell senescence and aging. J Clin Invest 132(13):115 10.1172/JCI158447 PMC9246372 35775483

[CR148] Miyasaki JM, Shannon K, Voon V, Ravina B, Kleiner-Fisman G, Anderson K, Shulman LM, Gronseth G, Weiner WJ (2006) Practice parameter: evaluation and treatment of depression, psychosis, and dementia in Parkinson disease (an evidence-based review): report of the Quality Standards Subcommittee of the American Academy of Neurology. Neurology 66(7):996–1002. 10.1212/01.wnl.0000215428.46057.3d 16606910 10.1212/01.wnl.0000215428.46057.3d

[CR149] Morris RG (2003) Long-term potentiation and memory. Philos Trans R Soc Lond B Biol Sci 358(1432):643–647 12740109 10.1098/rstb.2002.1230 PMC1693171

[CR150] Mozaffarian D, Benjamin EJ, Go AS, Arnett DK, Blaha MJ, Cushman M, de Ferranti S, Després JP, Fullerton HJ, Howard VJ, Huffman MD, Judd SE, Kissela BM, Lackland DT, Lichtman JH, Lisabeth LD, Liu S, Mackey RH, Matchar DB, Turner MB (2015) Heart disease and stroke statistics–2015 update: a report from the American heart association. Circulation 131(4):e29-322. 10.1161/cir.0000000000000152 25520374 10.1161/CIR.0000000000000152

[CR151] Naaz S, Mishra S, Pal PK, Chattopadhyay A, Das AR, Bandyopadhyay D (2020) Activation of SIRT1/PGC 1α/SIRT3 pathway by melatonin provides protection against mitochondrial dysfunction in isoproterenol induced myocardial injury. Heliyon 6 (10) 10.1016/j.heliyon.2020.e05159 PMC7567935 33088945

[CR152] Nadri P, Zahmatkesh A, Bakhtari A (2024) The potential effect of melatonin on in vitro oocyte maturation and embryo development in animals. Biol Reprod 111(3):529–542 38753882 10.1093/biolre/ioae077

[CR245] Ng KY, Leong MK, Liang H, Paxinos G (2017) Melatonin receptors: distribution in mammalian brain and their respective putative functions. Brain Struct Funct 222(7):2921–2939. 10.1007/s00429-017-1439-6 28478550 10.1007/s00429-017-1439-6

[CR153] Ni C, Tan G, Luo A, Qian M, Tang Y, Zhou Y, Wang J, Li M, Zhang Y, Jia D, Wu C, Guo X (2013) Melatonin premedication attenuates isoflurane anesthesia-induced β-amyloid generation and cholinergic dysfunction in the hippocampus of aged rats. Int J Neurosci 123(4):213–220. 10.3109/00207454.2012.742895 23256744 10.3109/00207454.2012.742895

[CR154] Nicolas A, Kenna KP, Renton AE, Ticozzi N, Faghri F, Chia R, Dominov JA, Kenna BJ, Nalls MA, Keagle P, Rivera AM, van Rheenen W, Murphy NA, van Vugt J, Geiger JT, Van der Spek RA, Pliner HA, Shankaracharya SBN, Landers JE (2018) Genome-wide analyses identify KIF5A as a novel ALS gene. Neuron 97(6):1267–1288. 10.1016/j.neuron.2018.02.027 29566793 10.1016/j.neuron.2018.02.027 PMC5867896

[CR155] Nikolaev G, Robeva R, Konakchieva R (2021) Membrane melatonin receptors activated cell signaling in physiology and disease. Int J Mol Sci 23(1):471 35008896 10.3390/ijms23010471 PMC8745360

[CR156] Northrop JH (1925) The influence of the intensity of light on the rate of growth and duration of life of drosophila. J Gen Physiol 9(1):81–86. 10.1085/jgp.9.1.81 19872234 10.1085/jgp.9.1.81 PMC2140775

[CR244] Nosjean O, Ferro M, Coge F, Beauverger P, Henlin JM, Lefoulon F, Fauchere JL, Delagrange P, Canet E, Boutin JA (2000) Identification of the melatonin-binding site MT3 as the quinone reductase 2. J Biol Chem 275(40):31311–31317. 10.1074/jbc.M005141200 10913150 10.1074/jbc.M005141200

[CR157] Okatani Y, Wakatsuki A, Reiter RJ, Enzan H, Miyahara Y (2003) Protective effect of melatonin against mitochondrial injury induced by ischemia and reperfusion of rat liver. Eur J Pharmacol 469(1–3):145–152. 10.1016/s0014-2999(03)01643-1 12782196 10.1016/s0014-2999(03)01643-1

[CR158] Okeke ES, Ogugofor MO, Nkwoemeka NE, Nweze EJ, Okoye CO (2022) Phytomelatonin: a potential phytotherapeutic intervention on COVID-19-exposed individuals. Microbes Infect 24(1):104886. 10.1016/j.micinf.2021.104886 34534695 10.1016/j.micinf.2021.104886 PMC8440003

[CR159] Pandi-Perumal SR, BaHammam AS, Brown GM, Spence DW, Bharti VK, Kaur C, Hardeland R, Cardinali DP (2013) Melatonin antioxidative defense: therapeutical implications for aging and neurodegenerative processes. Neurotox Res 23(3):267–300. 10.1007/s12640-012-9337-4 22739839 10.1007/s12640-012-9337-4

[CR160] Pannu A, Goyal RK (2024) From evidence to practice: A comprehensive analysis of side effects in synthetic anti-depressant therapy. Curr Drug Saf 19:10 10.2174/0115748863301630240417071353 38676478

[CR161] Parada E, Buendia I, León R, Negredo P, Romero A, Cuadrado A, López MG, Egea J (2014) Neuroprotective effect of melatonin against ischemia is partially mediated by alpha-7 nicotinic receptor modulation and HO-1 overexpression. J Pineal Res 56(2):204–212. 10.1111/jpi.12113 24350834 10.1111/jpi.12113

[CR162] Paulis L, Simko F, Laudon M (2012) Cardiovascular effects of melatonin receptor agonists. Expert Opin Investig Drugs 21(11):1661–1678. 10.1517/13543784.2012.714771 22916799 10.1517/13543784.2012.714771

[CR163] Paulose JK, Wright JM, Patel AG, Cassone VM (2016) Human gut bacteria are sensitive to melatonin and express endogenous circadian rhythmicity. PLoS ONE 11(1):e0146643. 10.1371/journal.pone.0146643 26751389 10.1371/journal.pone.0146643 PMC4709092

[CR164] Perry E, McKeith I, Ballard C (2003) Butyrylcholinesterase and progression of cognitive deficits in dementia with Lewy bodies. Neurology 60(11):1852–1853. 10.1212/01.wnl.0000068336.84399.9e 12796550 10.1212/01.wnl.0000068336.84399.9e

[CR165] Piemontese L, Tomás D, Hiremathad A, Capriati V, Candeias E, Cardoso SM, Chaves S, Santos MA (2018) Donepezil structure-based hybrids as potential multifunctional anti-Alzheimer’s drug candidates. J Enzyme Inhib Med Chem 33(1):1212–1224. 10.1080/14756366.2018.1491564 30160188 10.1080/14756366.2018.1491564 PMC6127844

[CR166] Pierpaoli W (1991) The pineal control of aging. The effects of melatonin and pineal grafting on the survival of older mice. Ann N Y Acad Sci 621:291–313. 10.1111/j.1749-6632.1991.tb16987.x 1859093 10.1111/j.1749-6632.1991.tb16987.x

[CR167] Poeggeler B, Reiter RJ, Tan DX, Chen LD, Manchester LC (1993) Melatonin, hydroxyl radical-mediated oxidative damage, and aging: a hypothesis. J Pineal Res 14(4):151–168. 10.1111/j.1600-079x.1993.tb00498.x 8102180 10.1111/j.1600-079x.1993.tb00498.x

[CR168] Poeggeler B, Sambamurti K, Siedlak SL, Perry G, Smith MA, Pappolla MA (2010) A novel endogenous indole protects rodent mitochondria and extends rotifer lifespan. PLoS ONE 5(4):e10206. 10.1371/journal.pone.0010206 20421998 10.1371/journal.pone.0010206 PMC2858081

[CR169] Quinn J, Kulhanek D, Nowlin J, Jones R, Praticò D, Rokach J, Stackman R (2005) Chronic melatonin therapy fails to alter amyloid burden or oxidative damage in old Tg2576 mice: implications for clinical trials. Brain Res 1037(1):209–213. 10.1016/j.brainres.2005.01.023 15777772 10.1016/j.brainres.2005.01.023

[CR170] Radak Z, Zhao Z, Goto S, Koltai E (2011) Age-associated neurodegeneration and oxidative damage to lipids, proteins and DNA. Mol Aspects Med 32(4–6):305–315 22020115 10.1016/j.mam.2011.10.010

[CR171] Ramírez-Rodríguez G, Klempin F, Babu H, Benítez-King G, Kempermann G (2009) Melatonin modulates cell survival of new neurons in the hippocampus of adult mice. Neuropsychopharmacology 34(9):2180–2191. 10.1038/npp.2009.46 19421166 10.1038/npp.2009.46

[CR172] Rashid I, Mir MA, Andleeb A, Kumar A, Munshi U, Habib D, Ahmad S (2024) Role of melatonin receptors as regulators of neurophysiology and therapeutic targets. J Pharma Insights Res 2(2):255–265

[CR173] Reina M, Martínez A (2018) A new free radical scavenging cascade involving melatonin and three of its metabolites (3OHM, AFMK and AMK). Comput Theor Chem 1123:111–118

[CR174] Reingold JL, Morgan JC, Sethi KD (2007) Rivastigmine for the treatment of dementia associated with Parkinson’s disease. Neuropsychiatr Dis Treat 3(6):775–783. 10.2147/ndt.s1134 19300613 10.2147/ndt.s1134 PMC2656320

[CR175] Reiter RJ (1980) The pineal and its hormones in the control of reproduction in mammals. Endocr Rev 1(2):109–131. 10.1210/edrv-1-2-109 6263600 10.1210/edrv-1-2-109

[CR176] Reiter RJ (1992) The ageing pineal gland and its physiological consequences. BioEssays 14(3):169–175. 10.1002/bies.950140307 1586370 10.1002/bies.950140307

[CR177] Reiter RJ, Tan DX, Mayo JC, Sainz RM, Lopez-Burillo S (2002) Melatonin, longevity and health in the aged: an assessment. Free Radic Res 36(12):1323–1329. 10.1080/1071576021000038504 12607824 10.1080/1071576021000038504

[CR178] Reiter RJ, Rosales-Corral SA, Tan DX, Alatorre-Jimenez M, Lopez C (2017) Circadian dysregulation and melatonin rhythm suppression in the context of aging. Circadian rhythms and their impact on aging:1–25

[CR179] Riemann D, Benz F, Dressle RJ, Espie CA, Johann AF, Blanken TF, Leerssen J, Wassing R, Henry AL, Kyle SD (2022) Insomnia disorder: state of the science and challenges for the future. J Sleep Res 31(4):e13604 35460140 10.1111/jsr.13604

[CR180] Rison RA, Stanton PK (1995) Long-term potentiation and N-methyl-D-aspartate receptors: foundations of memory and neurologic disease? Neurosci Biobehav Rev 19(4):533–552. 10.1016/0149-7634(95)00017-8 8684715 10.1016/0149-7634(95)00017-8

[CR181] Rossi SP, Matzkin ME, Riviere E, Martinez G, Ponzio R, Levalle O, Terradas C, Calandra RS, Frungieri MB (2023) Melatonin improves oxidative state and lactate metabolism in rodent Sertoli cells. Mol Cell Endocrinol 576:112034. 10.1016/j.mce.2023.112034 37516434 10.1016/j.mce.2023.112034

[CR248] Rossotti FJC, Rossotti H (1965) Potentiometric titrations using Gran plots: a textbook omission. J Chem Educ 42(7):375. 10.1021/ed042p375

[CR182] Roy K, Chakrabarti O, Mukhopadhyay D (2014) Interaction of Grb2 SH3 domain with UVRAG in an Alzheimer’s disease-like scenario. Biochem Cell Biol 92(3):219–225. 10.1139/bcb-2014-0001 24882360 10.1139/bcb-2014-0001

[CR183] Roy J, Tsui KC, Ng J, Fung ML, Lim LW (2021) Regulation of melatonin and neurotransmission in Alzheimer’s disease. Int J Mol Sci 22(13):6841 34202125 10.3390/ijms22136841 PMC8268832

[CR184] Roy J, Wong KY, Aquili L, Uddin MS, Heng BC, Tipoe GL, Wong KH, Fung ML, Lim LW (2022) Role of melatonin in Alzheimer’s disease: From preclinical studies to novel melatonin-based therapies. Front Neuroendocrinol 65:100986. 10.1016/j.yfrne.2022.100986 35167824 10.1016/j.yfrne.2022.100986

[CR185] Rudnick ND, Griffey CJ, Guarnieri P, Gerbino V, Wang X, Piersaint JA, Tapia JC, Rich MM, Maniatis T (2017) Distinct roles for motor neuron autophagy early and late in the SOD1(G93A) mouse model of ALS. Proc Natl Acad Sci USA 114(39):E8294-e8303. 10.1073/pnas.1704294114 28904095 10.1073/pnas.1704294114 PMC5625902

[CR186] Samanta S (2022) Physiological and pharmacological perspectives of melatonin. Arch Physiol Biochem 128(5):1346–1367 32520581 10.1080/13813455.2020.1770799

[CR187] Sampietro A, Pérez-Areales FJ, Martínez P, Arce EM, Galdeano C, Muñoz-Torrero D (2022) Unveiling the multitarget anti-alzheimer drug discovery landscape: a bibliometric analysis. Pharmaceuticals (Basel). 10.3390/ph15050545 35631371 10.3390/ph15050545 PMC9146451

[CR188] Sarlak G, Jenwitheesuk A, Chetsawang B, Govitrapong P (2013) Effects of melatonin on nervous system aging: neurogenesis and neurodegeneration. J Pharmacol Sci 123(1):9–24 23985544 10.1254/jphs.13r01sr

[CR189] Schieber M, Chandel NS (2014) ROS function in redox signaling and oxidative stress. Curr Biol 24(10):R453-462. 10.1016/j.cub.2014.03.034 24845678 10.1016/j.cub.2014.03.034 PMC4055301

[CR190] Scrivo A, Bourdenx M, Pampliega O, Cuervo AM (2018) Selective autophagy as a potential therapeutic target for neurodegenerative disorders. Lancet Neurol 17(9):802–815. 10.1016/s1474-4422(18)30238-2 30129476 10.1016/S1474-4422(18)30238-2 PMC6359907

[CR191] Shen D, Tian X, Sang W, Song R (2016) Effect of melatonin and resveratrol against memory impairment and hippocampal damage in a rat model of vascular dementia. NeuroImmunoModulation 23(5–6):318–331. 10.1159/000454681 28419991 10.1159/000454681

[CR192] Shi Y, Fang Y-Y, Wei Y-P, Jiang Q, Zeng P, Tang N, Lu Y, Tian Q (2018) Melatonin in synaptic impairments of Alzheimer’s disease. J Alzheimer’s Dis 63(3):911–926 29710712 10.3233/JAD-171178

[CR193] Sies H, Jones DP (2020) Reactive oxygen species (ROS) as pleiotropic physiological signalling agents. Nat Rev Mol Cell Biol 21(7):363–383. 10.1038/s41580-020-0230-3 32231263 10.1038/s41580-020-0230-3

[CR194] Simonsen A, Cumming RC, Brech A, Isakson P, Schubert DR, Finley KD (2008) Promoting basal levels of autophagy in the nervous system enhances longevity and oxidant resistance in adult Drosophila. Autophagy 4(2):176–184. 10.4161/auto.5269 18059160 10.4161/auto.5269

[CR195] Singh S, Rana A, Kaur S, Singh J, Rahi V, Choudhury H, Kumar P (2020) Pharmacology of melatonin and its receptors. Front Pharmacol Neurotrans 20:293–324

[CR196] Skarlis C, Anagnostouli M (2020) The role of melatonin in multiple sclerosis. Neurol Sci 41(4):769–781. 10.1007/s10072-019-04137-2 31845043 10.1007/s10072-019-04137-2

[CR197] Song Y, Yoon M (2025) Melatonin effects on animal behavior: circadian rhythm, stress response, and modulation of behavioral patterns. J Anim Sci Technol 67(1):1 39974791 10.5187/jast.2024.e105 PMC11833209

[CR198] Spencer CM, Noble S (1998) Rivastigmine. A review of its use in Alzheimer’s disease. Drugs Aging 13(5):391–411. 10.2165/00002512-199813050-00005 9829166 10.2165/00002512-199813050-00005

[CR199] Su SC, Hsieh MJ, Yang WE, Chung WH, Reiter RJ, Yang SF (2017) Cancer metastasis: mechanisms of inhibition by melatonin. J Pineal Res 62(1):e12370 10.1111/jpi.12370 27706852

[CR200] Sugumaran R, Krishna KSS, Saibaba J, Narayan SK, Sandhiya S, Rajeswari M (2024) Melatonin on sleep in Parkinson’s disease: a randomized double blind placebo controlled trial. Sleep Med 124:502–509 39437460 10.1016/j.sleep.2024.10.020

[CR201] Sun C, Qiu X, Wang Y, Liu J, Li Q, Jiang H, Li S, Song C (2020) Long-term oral melatonin alleviates memory deficits, reduces amyloid-β deposition associated with downregulation of BACE1 and mitophagy in APP/PS1 transgenic mice. Neurosci Lett 735:135192. 10.1016/j.neulet.2020.135192 32619650 10.1016/j.neulet.2020.135192

[CR202] Takahashi Y, Okada T (2011) Involvement of the nitric oxide cascade in melatonin-induced inhibition of long-term potentiation at hippocampal CA1 synapses. Neurosci Res 69(1):1–7. 10.1016/j.neures.2010.09.004 20875465 10.1016/j.neures.2010.09.004

[CR203] Talpur H, Chandio I, Brohi R, Worku T, Rehman Z, Bhattarai D, Ullah F, JiaJia L, Yang L (2018) Research progress on the role of melatonin and its receptors in animal reproduction: a comprehensive review. Reprod Domest Anim 53(4):831–849 29663591 10.1111/rda.13188

[CR204] Tan DX, Manchester LC, Reiter RJ, Qi WB, Karbownik M, Calvo JR (2000) Significance of melatonin in antioxidative defense system: reactions and products. Biol Signals Recept 9(3–4):137–159. 10.1159/000014635 10899700 10.1159/000014635

[CR205] Tchekalarova J, Georgieva I, Vukova T, Apostolova S, Tzoneva R (2025) Pinealectomy-induced melatonin deficiency exerts age-specific effects on sphingolipid turnover in rats. Int J Mol Sci 26(4):1694 40004158 10.3390/ijms26041694 PMC11855455

[CR206] Tripathy S, Bhattamisra SK (2025) Cellular signalling of melatonin and its role in metabolic disorders. Mol Biol Rep 52(1):1–12 10.1007/s11033-025-10306-8 39903334

[CR207] Uddin MS, Tewari D, Al Mamun A, Kabir MT, Niaz K, Wahed MII, Barreto GE, Ashraf GM (2020) Circadian and sleep dysfunction in Alzheimer’s disease. Ageing Res Rev 60:101046 32171783 10.1016/j.arr.2020.101046

[CR208] Van Acker ZP, Bretou M, Annaert W (2019) Endo-lysosomal dysregulations and late-onset Alzheimer’s disease: impact of genetic risk factors. Mol Neurodegener 14(1):20. 10.1186/s13024-019-0323-7 31159836 10.1186/s13024-019-0323-7 PMC6547588

[CR209] van Deursen JM (2019) Senolytic therapies for healthy longevity. Science 364(6441):636–637 31097655 10.1126/science.aaw1299 PMC6816502

[CR210] Verma AK, Singh S, Rizvi SI (2021) Age-dependent effect of continuous ‘artificial light at night’ on circadian rhythm in male rats: neuroprotective role of melatonin. Biogerontology 22(5):531–545. 10.1007/s10522-021-09933-y 34468927 10.1007/s10522-021-09933-y

[CR211] Verma AK, Singh S, Rizvi SI (2023) Therapeutic potential of melatonin and its derivatives in aging and neurodegenerative diseases. Biogerontology 24(2):183–206. 10.1007/s10522-022-10006-x 36550377 10.1007/s10522-022-10006-x

[CR212] Vicente-Zurdo D, Rosales-Conrado N, León-González ME, Brunetti L, Piemontese L, Pereira-Santos AR, Cardoso SM, Madrid Y, Chaves S, Santos MA (2022) Novel rivastigmine derivatives as promising multi-target compounds for potential treatment of Alzheimer’s disease. Biomedicines. 10.3390/biomedicines10071510 35884815 10.3390/biomedicines10071510 PMC9313321

[CR247] Videnovic A, Noble C, Reid KJ, Peng J, Turek FW, Marconi A, Rademaker AW, Simuni T, Zadikoff C, Zee PC (2014) Circadian melatonin rhythm and excessive daytime sleepiness in Parkinson disease. JAMA Neurol 71(4):463–469. 10.1001/jamaneurol.2013.6239 24566763 10.1001/jamaneurol.2013.6239 PMC4078989

[CR213] Walker WH, Walton JC, DeVries AC, Nelson RJ (2020) Circadian rhythm disruption and mental health. Transl Psychiatry 10(1):28 32066704 10.1038/s41398-020-0694-0 PMC7026420

[CR214] Wang LM, Suthana NA, Chaudhury D, Weaver DR, Colwell CS (2005) Melatonin inhibits hippocampal long-term potentiation. Eur J Neurosci 22(9):2231–2237. 10.1111/j.1460-9568.2005.04408.x 16262661 10.1111/j.1460-9568.2005.04408.x PMC2581482

[CR215] Wang X, Sirianni A, Pei Z, Cormier K, Smith K, Jiang J, Zhou S, Wang H, Zhao R, Yano H, Kim JE, Li W, Kristal BS, Ferrante RJ, Friedlander RM (2011) The melatonin MT1 receptor axis modulates mutant Huntingtin-mediated toxicity. J Neurosci 31(41):14496–14507. 10.1523/jneurosci.3059-11.2011 21994366 10.1523/JNEUROSCI.3059-11.2011 PMC3213696

[CR216] Wang DD, Jin MF, Zhao DJ, Ni H (2019a) Reduction of mitophagy-related oxidative stress and preservation of mitochondria function using melatonin therapy in an HT22 hippocampal neuronal cell model of glutamate-induced excitotoxicity. Front Endocrinol (Lausanne) 10:550. 10.3389/fendo.2019.00550 31440210 10.3389/fendo.2019.00550 PMC6694460

[CR217] Wang J, Wang X, He Y, Jia L, Yang CS, Reiter RJ, Zhang J (2019b) Antioxidant and Pro-Oxidant Activities of Melatonin in the Presence of Copper and Polyphenols In Vitro and In Vivo. Cells 8(8):903. 10.3390/cells8080903 31443259 10.3390/cells8080903 PMC6721667

[CR218] Wang YQ, Jiang YJ, Zou MS, Liu J, Zhao HQ, Wang YH (2022) Antidepressant actions of melatonin and melatonin receptor agonist: focus on pathophysiology and treatment. Behav Brain Res 420:113724 34929236 10.1016/j.bbr.2021.113724

[CR219] Wani A, Gupta M, Ahmad M, Shah AM, Ahsan AU, Qazi PH, Malik F, Singh G, Sharma PR, Kaddoumi A, Bharate SB, Vishwakarma RA, Kumar A (2019) Alborixin clears amyloid-β by inducing autophagy through PTEN-mediated inhibition of the AKT pathway. Autophagy 15(10):1810–1828. 10.1080/15548627.2019.1596476 30894052 10.1080/15548627.2019.1596476 PMC6735498

[CR220] Watabe N, Ishida Y, Ochiai A, Tokuoka Y, Kawashima N (2007) Oxidation decomposition of unsaturated fatty acids by singlet oxygen in phospholipid bilayer membranes. J Oleo Sci 56(2):73–80. 10.5650/jos.56.73 17898466 10.5650/jos.56.73

[CR221] Weil ZM, Hotchkiss AK, Gatien ML, Pieke-Dahl S, Nelson RJ (2006) Melatonin receptor (MT1) knockout mice display depression-like behaviors and deficits in sensorimotor gating. Brain Res Bull 68(6):425–429. 10.1016/j.brainresbull.2005.09.016 16459197 10.1016/j.brainresbull.2005.09.016

[CR222] Wilhelm T, Byrne J, Medina R, Kolundžić E, Geisinger J, Hajduskova M, Tursun B, Richly H (2017) Neuronal inhibition of the autophagy nucleation complex extends life span in post-reproductive C. elegans. Genes Dev 31(15):1561–1572. 10.1101/gad.301648.117 28882853 10.1101/gad.301648.117 PMC5630021

[CR223] Wilkerson HL (1947) Problems of an aging population; public health aspects of diabetes. Am J Public Health Nations Health 37(2):177–188 18016480 PMC1623303

[CR224] Wu YH, Swaab DF (2005) The human pineal gland and melatonin in aging and Alzheimer’s disease. J Pineal Res 38(3):145–152. 10.1111/j.1600-079X.2004.00196.x 15725334 10.1111/j.1600-079X.2004.00196.x

[CR225] Wu YH, Swaab DF (2007) Disturbance and strategies for reactivation of the circadian rhythm system in aging and Alzheimer’s disease. Sleep Med 8(6):623–636. 10.1016/j.sleep.2006.11.010 17383938 10.1016/j.sleep.2006.11.010

[CR226] Wu YH, Ursinus J, Zhou JN, Scheer FA, Ai-Min B, Jockers R, van Heerikhuize J, Swaab DF (2013) Alterations of melatonin receptors MT1 and MT2 in the hypothalamic suprachiasmatic nucleus during depression. J Affect Disord 148(2–3):357–367. 10.1016/j.jad.2012.12.025 23357659 10.1016/j.jad.2012.12.025

[CR227] Xia Z, Storm D (2017) Role of circadian rhythm and REM sleep for memory consolidation. Neurosci Res 118:13–20 28434990 10.1016/j.neures.2017.04.011 PMC8051942

[CR228] Xie Z, Chen F, Li WA, Geng X, Li C, Meng X, Feng Y, Liu W, Yu F (2017) A review of sleep disorders and melatonin. Neurol Res 39(6):559–565 28460563 10.1080/01616412.2017.1315864

[CR229] Xu H, Chen L, Zhang X, Jiang X, Tian W, Yu W, Wang X, Tian J, Su D (2019a) Central cholinergic neuronal degeneration promotes the development of postoperative cognitive dysfunction. Lab Invest 99(7):1078–1088. 10.1038/s41374-018-0174-9 30626892 10.1038/s41374-018-0174-9

[CR230] Xu J, Gao H, Zhang L, Rong S, Yang W, Ma C, Chen M, Huang Q, Deng Q, Huang F (2019b) Melatonin alleviates cognition impairment by antagonizing brain insulin resistance in aged rats fed a high-fat diet. J Pineal Res 67(2):e12584 31050371 10.1111/jpi.12584

[CR231] Xu H, Liu YY, Li LS, Liu YS (2023) Sirtuins at the crossroads between mitochondrial quality control and neurodegenerative diseases: structure, regulation, modifications, and modulators. Aging Dis 14(3):794 37191431 10.14336/AD.2022.1123 PMC10187712

[CR232] Yan M, Sun S, Xu K, Huang X, Dou L, Pang J, Tang W, Shen T, Li J (2021) Cardiac aging: from basic research to therapeutics. Oxid Med Cell Longev 2021:9570325. 10.1155/2021/9570325 33777324 10.1155/2021/9570325 PMC7969106

[CR233] Yang G, Chen L, Grant GR, Paschos G, Song WL, Musiek ES, Lee V, McLoughlin SC, Grosser T, Cotsarelis G, FitzGerald GA (2016) Timing of expression of the core clock gene Bmal1 influences its effects on aging and survival. Sci Transl Med 8(324):324316. 10.1126/scitranslmed.aad3305 10.1126/scitranslmed.aad3305 PMC4870001 26843191

[CR234] Yu C (2021) Xiao J-H (2021) The Keap1-Nrf2 system: a mediator between oxidative stress and aging. Oxid Med Cell Longev 1:6635460 10.1155/2021/6635460 PMC8106771 34012501

[CR235] Zhang W, Xiong BR, Zhang LQ, Huang X, Zhou WC, Zou Q, Manyande A, Wang J, Tian XB, Tian YK (2020) Disruption of the GABAergic system contributes to the development of perioperative neurocognitive disorders after anesthesia and surgery in aged mice. CNS Neurosci Ther 26(9):913–924. 10.1111/cns.13388 32488976 10.1111/cns.13388 PMC7415208

[CR236] Zhang C, Lv Y, Bai R, Xie Y (2021a) Structural exploration of multifunctional monoamine oxidase B inhibitors as potential drug candidates against Alzheimer’s disease. Bioorg Chem 114:105070. 10.1016/j.bioorg.2021.105070 34126574 10.1016/j.bioorg.2021.105070

[CR237] Zhang Y, Zhang J, Wang S (2021b) The role of rapamycin in healthspan extension via the delay of organ aging. Ageing Res Rev 70:101376 34089901 10.1016/j.arr.2021.101376

[CR238] Zhang C, Ma Y, Zhao Y, Guo N, Han C, Wu Q, Mu C, Zhang Y, Tan S, Zhang J (2024) Systematic review of melatonin in cerebral ischemia-reperfusion injury: critical role and therapeutic opportunities. Front Pharmacol 15:1356112 38375039 10.3389/fphar.2024.1356112 PMC10875093

[CR239] Zhang Z, Xue P, Bendlin BB, Zetterberg H, De Felice F, Tan X, Benedict C (2025) Melatonin: a potential nighttime guardian against Alzheimer’s. Mol Psychiatry 30(1):237–250. 10.1038/s41380-024-02691-6 39128995 10.1038/s41380-024-02691-6 PMC11649572

[CR240] Zhao H, Li L, Zhang X, Shi J, Lai W, Wang W, Guo L, Gong J, Lu C (2024) Global, regional, and national burden of depressive disorders among young people aged 10–24 years, 2010–2019. J Psychiatr Res 170:47–57 38103449 10.1016/j.jpsychires.2023.11.047

[CR241] Zhu L, Zee PC (2012) Circadian rhythm sleep disorders. Neurol Clin 30(4):1167–1191. 10.1016/j.ncl.2012.08.011 23099133 10.1016/j.ncl.2012.08.011 PMC3523094

[CR242] Zhu Y, Runwal G, Obrocki P, Rubinsztein DC (2019) Autophagy in childhood neurological disorders. Dev Med Child Neurol 61(6):639–645. 10.1111/dmcn.14092 30417343 10.1111/dmcn.14092

[CR243] Zisapel N (2018) New perspectives on the role of melatonin in human sleep, circadian rhythms and their regulation. Br J Pharmacol 175(16):3190–3199. 10.1111/bph.14116 29318587 10.1111/bph.14116 PMC6057895

